# WNT signaling inducing activity in ascites predicts poor outcome in ovarian cancer

**DOI:** 10.7150/thno.37423

**Published:** 2020-01-01

**Authors:** Anna Kotrbová, Petra Ovesná, Tomáš Gybel', Tomasz Radaszkiewicz, Markéta Bednaříková, Jitka Hausnerová, Eva Jandáková, Luboš Minář, Igor Crha, Vít Weinberger, Luděk Záveský, Vítězslav Bryja, Vendula Pospíchalová

**Affiliations:** 1 Department of Experimental Biology, Faculty of Science, Masaryk University, Kotlářská 2, 611 37, Brno, Czech Republic; 2 Institute of Biostatistics and Analyses, Faculty of Medicine, Masaryk University, Kamenice 126/3, 625 00, Brno, Czech Republic; 3 Department of Internal Medicine - Hematology & Oncology, University Hospital Brno and Medical Faculty, Masaryk University, Jihlavská 20, 625 00, Brno, Czech Republic; 4 Department of Pathology, University Hospital Brno and Medical Faculty, Masaryk University, Jihlavská 20, 625 00, Brno, Czech Republic; 5 Department of Obstetrics and Gynecology, University Hospital Brno and Medical Faculty, Masaryk University, Jihlavská 20, 625 00, Brno, Czech Republic; 6 Department of Nursing and Midwifery, Faculty of Medicine, Masaryk University, Kamenice 753/5, 625 00, Brno, Czech Republic; 7 Institute of Biology and Medical Genetics, First Faculty of Medicine, Charles University and General University Hospital in Prague, Albertov 4, 128 00, Prague, Czech Republic

**Keywords:** ascites, casein kinase 1, high grade serous carcinoma of the ovary, fallopian tube and peritoneum, planar cell polarity pathway, WNT signaling

## Abstract

High grade serous carcinoma of the ovary, fallopian tube, and peritoneum (HGSC) is the deadliest gynecological disease which results in a five-year survival rate of 30% or less. HGSC is characterized by the early and rapid development of metastases accompanied by a high frequency of ascites *i.e.* the pathological accumulation of fluid in peritoneum. Ascites constitute a complex tumor microenvironment and contribute to disease progression by largely unknown mechanisms.

**Methods:** Malignant ascites obtained from HGSC patients who had undergone cytoreductive surgery were tested for their ability to induce WNT signaling in the Kuramochi cell line, a novel and clinically relevant *in vitro* model of HGSC. Next, cancer spheroids (the main form of metastatic cancer cells in ascites) were evaluated with respect to WNT signaling. Kuramochi cells were used to determine the role of individual WNT signaling branches in the adoption of metastatic stem cell-like behavior by HGSC cells. Furthermore, we analyzed genomic and transcriptomic data on WNT/Planar Cell Polarity (PCP) components retrieved from public cancer databases and corroborated with primary patient samples and validated antibodies on the protein level.

**Results:** We have shown that ascites are capable of inducing WNT signaling in primary HGSC cells and HGSC cell line, Kuramochi. Importantly, patients whose ascites cannot activate WNT pathway present with less aggressive disease and a considerably better outcome including overall survival (OS). Functionally, the activation of non-canonical WNT/PCP signaling by WNT5A (and not canonical WNT/β-catenin signaling by WNT3A) promoted the metastatic stem-cell (metSC) like behavior (*i.e.* self-renewal, migration, and invasion) of HGSC cells. The pharmacological inhibition of casein kinase 1 (CK1) as well as genetic ablation (dishevelled 3 knock out) of the pathway blocked the WNT5A-induced effect. Additionally, WNT/PCP pathway components were differentially expressed between healthy and tumor tissue as well as between the primary tumor and metastases. Additionally, ascites which activated WNT/PCP signaling contained the typical WNT/PCP ligand WNT5A and interestingly, patients with high levels of WNT5A protein in their ascites exhibited poor progression-free survival (PFS) and OS in comparison to patients with low or undetectable ascitic WNT5A. Together, our results suggest the existence of a positive feedback loop between tumor cells producing WNT ligands and ascites that distribute WNT activity to cancer cells in the peritoneum, in order to promote their pro-metastatic features and drive HGSC progression.

**Conclusions:** Our results highlight the role of WNT/PCP signaling in ovarian cancerogenesis, indicate a possible therapeutic potential of CK1 inhibitors for HGSC, and strongly suggest that the detection of WNT pathway inducing activity ascites (or WNT5A levels in ascites as a surrogate marker) could be a novel prognostic tool for HGSC patients.

## Introduction

Ovarian cancer (OC) is the third leading gynecologic malignancy globally. However, it has the highest mortality rate of all gynecologic cancers accounting for nearly 185,000 deaths every year 1. The cause of most deaths (70%) has been attributed to aggressive high-grade serous carcinoma (HGSC) of the ovary, fallopian tube and peritoneum. The lack of screening methods in addition to the asymptomatic course of HGSC has resulted in late diagnosis after the primary tumor has already metastasized. Moreover, nearly 70% of ovarian cancer patients who experience successful treatment initially, inevitably develop chemoresistant disease 2 with patients falling into a 5-year survival of 26% (stage IV) and 42% (stage III) according to International Federation of Gynecology and Obstetrics (FIGO) staging for HGSC 3.

The general method of OC metastasis is transcoelomic spread. In this form of tumor dissemination, cancer cells are shed from tumors and then implant within the peritoneum. This process is often accompanied by the formation of ascites, a pathological accumulation of fluid which circulates inside the peritoneal cavity, also known as ascitic fluid, peritoneal fluid, or effusion. Ascites are believed to constitute a complex milieu and dynamic reservoir of factors that affect tumor cell growth and progression. HGSC patients often present with ascites at the time of diagnosis and have ascites therapeutically removed during cytoreductive surgery. Ascites can serve as an easily accessible sample that contains both cancer cells and their microenvironment 4. Thus, the molecular and functional analysis of ascites represents an undervalued source of information both for clinical diagnostics as well as the biological understanding of OC progression and resistance 5.

WNT signaling is a complex and fundamental developmental pathway which is known to be dysregulated in many types of human malignancies, including gynecologic cancers, which has been summarized in 6. WNT signaling is often regarded as one pathway, but it encompasses several distinct transduction cascades. WNT/β-catenin signaling constitutes the canonical pathway, which leads to transcriptional activation of target genes that are involved in proliferation and stem cell renewal (recently reviewed in 7). The WNT/Planar Cell Polarity (PCP) pathway represents the major branch of the non-canonical (or β-catenin independent) WNT pathways, which control cell polarity. WNT/PCP-mediated cell polarization is essential *e.g.* for asymmetric cell division or directional cell movement, functions critically involved in mammalian development and human cancerogenesis and metastatic processes 8.

In this study, we analyzed the ascites of HGSC patients for the ability to activate the WNT signaling pathway. We have shown that patient ascites can induce WNT signaling in HGSC cells which leads to a poor prognosis. We specified the activation of the non-canonical WNT pathway as the trigger that promotes migration, invasion, and stemness of HGSC cells. Finally, we have demonstrated that WNT5A is the source of WNT activity in ascites and that high levels of WNT5A protein in ascites are also sign of poor prognosis for HGSC patients.

## Materials and Methods

### Ethics statement

OC patient samples were collected at the Department of Obstetrics and Gynecology of University Hospital Brno, Czech Republic, under the written informed consent of patients and IRB protocol of Vitezslav Bryja (MUNI/M/1050/2013), Vendula Pospichalova (17-11776Y) and Ludek Zavesky (2060/11/S). The studies were approved by the Ethics Committee of University Hospital Brno and a multi-centric Ethics Committee of the General University Hospital in Prague. All specimens were handled according to ethical and legal standards. Complete clinicopathological data for each patient is available in Supplementary Table ST1.

### Ascites

Ascites were collected by oncogynecologists during cytoreductive surgery of HGSC patients, transported to laboratory at 4 °C and processed without undue delay. Each ascitic sample was centrifuged to remove cells (200 × g for 5 minutes) and apoptotic bodies (1,300 - 1,500 × g for 10 - 15 minutes), aliquoted and stored at -25 °C - -80 °C.

In total, fifty four ascitic fluid samples were tested for the ability to induce WNT signaling. Twenty four ascitic fluids were excluded from further analysis because of their cytotoxicity to Kuramochi cells even at 10% concentration (eight samples), non-HGSC histology (eleven samples) or due to treatment with olaparib (two samples).

Cancer spheroids were isolated from ascites according to a previously published protocol 9. In short, cells were pelleted at 500 × g for 5 minutes and red blood cells were lysed using ACK buffer. Remaining cells were washed in PBS and passed through a CellTricks 30 μm nylon filter (04-0042-2316, Sysmex). The filter was then washed with PBS. Spheroids which did not pass through the filter were backwashed off the filter with PBS, assessed for viability, and counted using trypan blue (17-942E, LONZA) staining and hemocytometer. Isolated spheroids were immediately used for experiments.

Fractionation of ascitic fluids was performed using a size exclusion chromatography column (Izon Science Ltd) according to manufacturer's instructions. Early fractions devoid of albumin were used for WB.

### Cell culture and treatment

The Kuramochi cell line (RRID:CVCL_1345) was purchased from JCRB Cell Bank and cultured in RPMI 1640 medium (SH30027, HyClone) supplemented with 10% FBS, 50 units/ml penicillin, and 50 units/ml streptomycin (15140122, Gibco). Ascitic spheroids were cultured in MCDB/DMEM (1:1) medium (10372019 and 31966047, Gibco) supplemented with 10% FBS, 50 units/ml penicillin, and 50 units/ml streptomycin on ultra-low attachment plates (Corning). Cells and spheroids were stimulated with recombinant WNT proteins (645-WN-010, 5036-WN-CF; RnD Systems) at a concentration of 200 ng/ml or by WNT conditioned medium (CM) (control, WNT3A or WNT5A), produced by rat L-fibroblasts (CRL-2648, CRL-2647, CRL-2814; ATCC), at a ratio of 1:4 with complete culture medium. The Porcupine inhibitor LGK974 (974-02; Stem RD) was used at a 0.1 μM concentration while CK1 inhibitors PF670462 [sc-204180A; Santa Cruz Biotechnology (SCBT)] and D4476 (218696; Calbiochem) were used in 5 μM final concentration. If not stated otherwise, cells were pretreated with either LGK974 (+), or DMSO (-) for 24-48 h and treated with recombinant human (rh) WNTs/WNT CM overnight before being used for assays or WB.

### Western blotting (WB)

WB and immunodetection was performed on protein lysates from tumor and healthy tissue samples as described previously 10. Protein lysates from cell culture experiments were prepared by direct lyses of samples (after PBS wash) in reducing Laemmli buffer as described in 11 with the exception of WNT5A detection, where the lysates were prepared in non-reducing Laemmli buffer. The antibodies used were rabbit anti β-ACTIN [cs-4970; Cell Signaling Technology (CST)], goat anti CELSR3 (sc-46849, SCBT), rabbit anti DVL2 (cs-3216; CST), mouse anti DVL3 (sc-8027; SCBT), rabbit anti FZD10 (ab71987, Abcam), rabbit anti LRP6 (cs-3395, CST), rabbit anti LRP6 phospho-S1490 (cs-2568, CST), rabbit anti PRICKLE1 (ab15577, Abcam), rabbit anti ROR1 (gift from Henry Ho 12), rabbit anti ROR2 (gift from Henry Ho 12), mouse anti ROR2 (sc-374174, SCBT), sheep anti VANGL2 (AF4815, RnD Systems), mouse anti α-TUBULIN (TG199, Sigma Aldrich), goat anti WNT5A (AF645, RnD Systems), rat anti WNT5A (MAB645, RnD Systems), rabbit anti WNT11 (LS-C185754, LifeSpan BioSciences). Beta-ACTIN and α-TUBULIN serve as loading control. A representative blot from one of three independent experiments is shown for each experiment, if not stated otherwise. Densitometry measurements and quantification was performed using ImageJ software. Quantification of WBs including statistical analyses of replicates is presented in Supplementary Figs. S1, S2, S5 and S7.

### Activation of WNT signaling by ascites

Kuramochi cells were pretreated with either LGK974 or DMSO and treated with non-cellular fractions of ascites at 10% - 50% concentration in culture medium overnight. If not otherwise stated, a 25% concentration was used. A representative blot from one of three to five independent experiments is shown. Active WNT signaling was assessed using a WB quantification of the ratio of phosphorylated (p) DVL to total DVL. WNT/β-catenin signaling was assessed as pLRP6/actin, while non-canonical WNT signaling as the electrophoretic shift of ROR receptors (pROR/total ROR). Densitometry was performed in ImageJ, WNT pathway activation was considered to be positive when *P* ≤ 0.05 (paired t-test) in comparison to the control.

### Activation of WNT signaling by WNT CM/rh WNTs

A representative blot from one of three independent experiments is shown. Active DVL2 and DVL3 were assessed by WB quantification (ratio of phosphorylated DVL to total DVL). Active WNT signaling was assessed by the WB quantification of pDVL/total DVL. WNT/β-catenin signaling was assessed as pLRP6/actin, while non-canonical WNT signaling as the electrophoretic shift of ROR receptors (pROR/total ROR). Densitometry was performed in ImageJ, WNT pathway activation was considered to be positive when *P* ≤ 0.05 (Tukey post-hoc test of one-way ANOVA) in comparison to the control.

### Luciferase assay

The activation of target genes of the canonical WNT pathway was assessed using Dual-Luciferase Reporter Assay System (E1960; Promega) and the TOPFLASH reporter according to manufacturer's instructions and as described previously 13.

### Migration/invasion assay

A migration/invasion assay was performed using a transwell system with 8.0 μm pores (CLS3384; Corning) using FBS as chemoattractant. For the invasion assay, membranes were pre-coated with 20 μg Matrigel Matrix (354234; Corning) prior to the experiment. Cells (10,000 cells per well for 96 well plate) were placed into the upper well in serum free medium (RPMI 1640 + 0.1% BSA + 50 units/ml penicillin and 50 units/ml streptomycin) while the lower well was filled with complete medium (RPMI 1640 + 10% FBS + 50 units/ml penicillin and 50 units/ml streptomycin). The migration assay ran for 4 h and invasion assay for 8 h. Afterwards the membranes were fixed in ice-cold methanol and stained with crystal violet (0.2 mg/ml crystal violet in 2% ethanol). Finally, the membranes were photographed under a microscope and the cells that migrated through the pores were counted using the ImageJ software counting tool.

### Real Time Cell Analysis (RTCA, xCELLigence system)

Quantitative cell migration analysis was performed using a CIM-plate 16 (5665817001, ACEA Biosciences, Inc.) with 8.0 μm pores and the xCELLigence RTCA DP Instrument (ACEA Biosciences, Inc.) according to manufacturer's instructions. Briefly, the lower chamber was filled with complete medium, upper chamber was attached to the lower chamber and filled with a small amount of serum free medium which contained 0.1% BSA. The assembled plate was placed into the xCELLigence RTCA DP Instrument (placed in cell culture incubator), 3 background sweeps were measured and then the plate was incubated for 1 h. Afterwards, cells (30,000 cells per well) were placed into the upper wells of the plate in serum free medium and left at room temperature for 30 min. Finally, the plate was placed into the xCELLigence RTCA DP instrument and measured every 15 min with 100 sweeps in total.

### Sphere forming assay

6,000 cells were grown in 1 ml of 1% methylcellulose in RPMI 1640 medium per well in a 24-well ultra-low attachment plate (CLS3473; Corning). The methylcellulose medium was supplemented with the following growth factors: 1 × B-27 supplement minus vitamin A (12587010; ThermoFisher Scientific), 0.02 μg/ml EGF (E9644; Sigma Aldrich), 0.02 μg/ml FGF2 (SRP4037; Sigma Aldrich), and 0.4 μg/ml human insulin (I9278; Sigma Aldrich). Cells were left to grow for 14 days. After this incubation period, any spheres larger than 50 µm were counted under microscope. The results were expressed as a sphere index, the value normalized to the mean of the DMSO triplicate control in each experiment.

### DVL3 KO Kuramochi cell line

DVL3-null Kuramochi cell lines were prepared according to a previously published protocol 14. Guide RNA had the following sequence: GCGAGAGCGGCCACGCCGG. PCR of genomic DNA was amplified with forward primer: TGACCTGAGGGTGGGGA and reverse primer: AAGGAGGGAGAGAGGCAAGC and yielded 349 bp product. After *Hpa*II restriction digestion of WT allele, two bands (185 bp and 164 bp) were generated. The PCR product from the mutant allele carrying the *Hpa*II recognition site disrupted by hCas9 mediated double strand break was not cut. Mutant cell clones were checked by WB using DVL3 antibodies sc-8027 (SCBT) or cs-3018 (CST) and by next generation sequencing using the Illumina platform as described previously 15. DVL3 KO clones as well as the parental Kuramochi cell line were verified using STR profiling authentication service.

### Measurement of cell proliferation

Cells were stained with 1.25 μM CFSE (65-0850-84; eBioscience, Inc.) and 10,000 cells per well were seeded directly to the medium containing 0.1 μM LGK974 and WNT CM in a ratio of 1:4. Each day, the contents of 1 well of the same condition was trypsinized and cell count was performed using a BD Accuri C6 (BD Biosciences) flow cytometer.

### Statistical analyses

All experiments were done in triplicates unless stated otherwise. Graphic illustrations were generated using Prism, version 8.0, GraphPad Software.

Data from the current COSMIC database, COSMIC v90, released 5^th^ September 2019 was implemented as well as data from the Oncomine database, Oncomine version v4.5. The Oncomine™ Platform (Thermo Fisher, Ann Arbor, MI) and Prism were used for analysis and visualization.

Statistical analysis of overall patient survival was performed on TCGA Ovarian dataset 16 retrieved from the Oncomine database. Only patients with primary occurrence of the tumor and primary tumor samples were included in the analysis. The Kaplan-Meier method was used for each possible cut-off of each gene probe survival curve. A log-rank test was then used to compare the difference between the groups (over and under the cut-off). The most significant cut-off was chosen for each gene probe. Possible prognostic gene probes (*i.e. P* < 0.05 of log-rank test) were then included in a multivariate Cox proportional hazard model to yield independent prognostic factors. Hazard ratios (HR), including 95% confidence intervals, were calculated. For genes with multiple probes, a gene was considered to be a possible prognostic factor when at least one probe reached *P* < 0.05 of log-rank test. Statistical analysis was performed in R software, version 3.2.5 (The R Foundation for Statistical Computing, Vienna, Austria; http://www.R-project.org/).

Data from the patient cohort from University Hospital Brno was analyzed using Prism (version 8.0) GraphPad Software. Both the Gehan-Breslow-Wilcoxon test (OS analysis) and log-rank test (Mantel-Cox) (PFS analysis) were used to measure the differences in the Kaplan-Meier curves.

### Meta-analysis in Oncomine

The Oncomine™ Platform (Thermo Fisher, Ann Arbor, MI) was used for analysis and visualization. Genes were arranged by median rank across the selected analyses and their corresponding *P* values were displayed. Individual analysis of gene rank was based on the gene's *P* value which was compared to all other genes within each analysis. In the heat map, red bars denote over-expression while blue bars denote under-expression. The color code indicates whether the individual gene rank is in top 1%, 5%, 10% or 25% of overexpressed/underexpressed genes. Dataset references can be seen in Supplementary Table ST4.

## Results

### Kuramochi is a suitable *in vitro* model for studying role of WNT signaling in progression of HGSC

**Figure 1:**
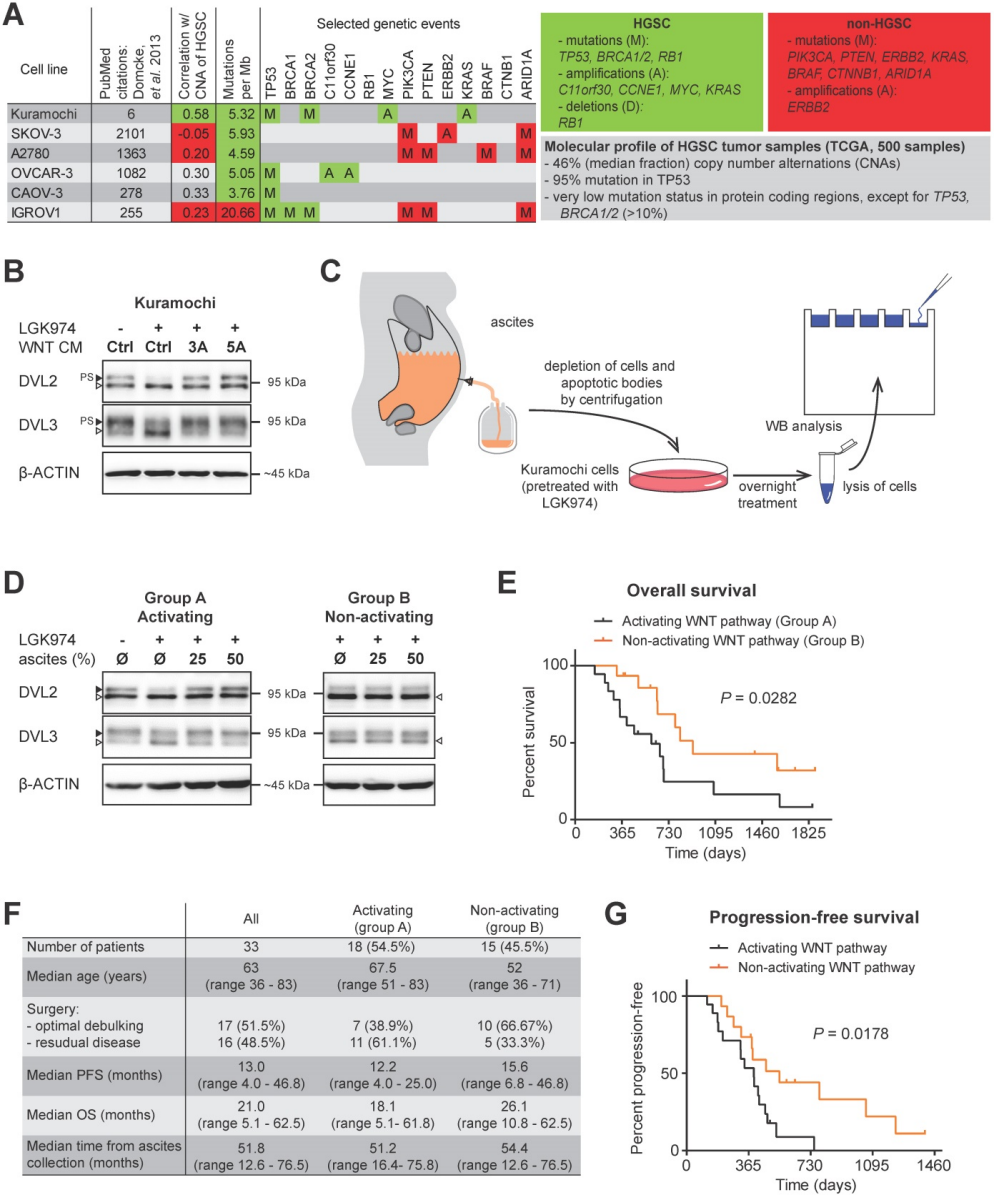
** Ascites can activate WNT signaling and this activity is associated with worse prognosis. A)** A synopsis of the evaluation of 47 OC cell lines used as HGSC tumor models by genomic profiling adapted from 17. Unlike the most frequently used cell lines (SKOV-3, A2780, OVCAR-3, CAOV-3 and IGROV1), the Kuramochi cell line credibly reflects HGSC both on the genomic and transcriptomic level. The grey panel summarizes the molecular profile of 500 HGSC tumor samples, the green panel summarizes the genetic events typical for HGSC and the red panel summarizes the genetic events typical for OC subtypes other than HGSC. **B)** Assessment of the WNT signaling induction in Kuramochi cell line. Active WNT signaling results in the phosphorylation-induced electrophoretic shift of DVL proteins. Black triangle - phosphorylated and shifted (PS-) form, open triangle - non-phosphorylated form. The endogenous expression of WNTs and subsequent autocrine stimulation is evidenced by the increase in PS-DVL to total levels of DVL protein in comparison with a control, LGK974 treated cells. Similarly, responsiveness to exogenous WNTs is shown. **C)** Scheme of the experiment used to assay WNT signaling activity in ascites. **D)** 54.5% of tested malignant ascites were able to induce WNT signaling in Kuramochi cells. Ascites were subsequently divided into two groups: A - WNT signaling activating ascites and B - WNT signaling non-activating ascites. **E)** Overall survival of patients in group A and B. **F)** Comparison of clinical characteristics and disease outcome of patients in groups A and B. **G)** Progression-free survival of patients in group A and B. Quantification of WBs is available in Supplementary Fig. S1.

The study of the complex molecular mechanisms of human diseases requires a suitable *in vitro* model that accurately represents a specific situation in patients. The extensive comparison of genomic and mRNA expression profiles of HGSC patients and the profiles of 47 OC cell lines revealed that the most frequently used cell lines poorly reflect the profiles seen in the HGSC samples 17 (Fig. 1A). Unfortunately, earlier studies focused primarily on the functional analysis of WNT signaling in OC using cell lines which may not be accurate, at least when compared to the molecular profile of HGSC 18-22. Therefore, we have performed our analyses with Kuramochi 23, the best scoring cell line with respect to HGSC molecular signature 17.

Ligand-induced activation of both the WNT/β-catenin and non-canonical WNT signaling pathway can be easily monitored exploiting cytoplasmic dishevelled (DVL) proteins. Hyperphosphorylation of DVL proteins (humans harbor 3 genes - *DVL1, DVL2* and *DVL3*) is the most general yet specific approach to monitor active WNT signaling 24. This hyperphosphorylation can be monitored by the electrophoretic mobility shift of DVL proteins by western blot (WB) analysis. Phosphorylated and shifted DVL (PS-DVL) is a hallmark of active WNT signaling. PS-DVL2 and PS-DVL3 levels were high in unstimulated Kuramochi cells (Fig. 1B, first line) which suggests the presence of an autocrine signaling loop. To test this assumption, we blocked the endogenous expression of WNT ligands by the inhibition of *O*-acyltransferase Porcupine, an enzyme that is required for the biogenesis of WNT ligands. Treatment of Kuramochi cells with LGK974, a specific inhibitor of Porcupine 25, reduced the levels of PS-DVL (Fig. 1B) in these cells. This phenotype could be restored with the treatment of exogenous WNT3A and WNT5A when provided in the form of CM 26, 27 (Fig. 1B, quantified in Supplementary Fig. S1A, S1B). Together, this data showed that Kuramochi cells both produce as well as respond to WNT ligands.

### Patients whose ascites are unable to induce WNT signaling exhibit better prognosis

Due to the lack of anatomical barriers surrounding the ovaries, HGSCs are easily capable of disseminating directly from the ovary/fallopian tube into the peritoneal cavity. This process is believed to be facilitated and/or mediated by ascites through different and largely unknown mechanisms 4. We therefore asked whether ascites could induce WNT signaling in HGSC cells. Ascitic samples were collected from fifty four patients during their surgical treatment for OC, non-cellular fractions of ascitic fluids were prepared and used for the treatment of Kuramochi cells (Fig. 1C). After a thorough examination of patient clinical data (see Materials and methods for details), a total of thirty three patients were included in the study. Eighteen patient ascites were able to activate WNT signaling, while fifteen ascites did not induce WNT signaling (Fig. 1D) as determined by the hyperphosphorylation of DVL2 and DVL3 proteins (for raw data see Supplementary Fig. S1C, S1D).

Therefore, we divided the patients' samples based on the ability of their ascites to trigger WNT pathway into two groups: A - WNT signaling activating ascites (N=18) and B - WNT signaling non-activating ascites (N=15). Intriguingly, when clinical parameters were compared, patients in group B whose ascites was unable to induce WNT signaling had better prognosis, including OS (median time of group A was 18.1 months, while group B was 26.1 months, *P* = 0.0281) (Fig. 1E, 1F). This was despite the comparable profile of major prognostic factors for HGSC - including surgical FIGO Stage (mostly IIIC), and residual tumor after surgery 28 - in both groups (Fig. 1F, Supplementary Table ST1). PFS was also significantly longer in group B patients than in group A patients (median 12.2 vs. 15.6 months) (Fig. 1F, 1G).

### Both canonical and non-canonical WNT signaling can be activated in HGSC cells

**Figure 2:**
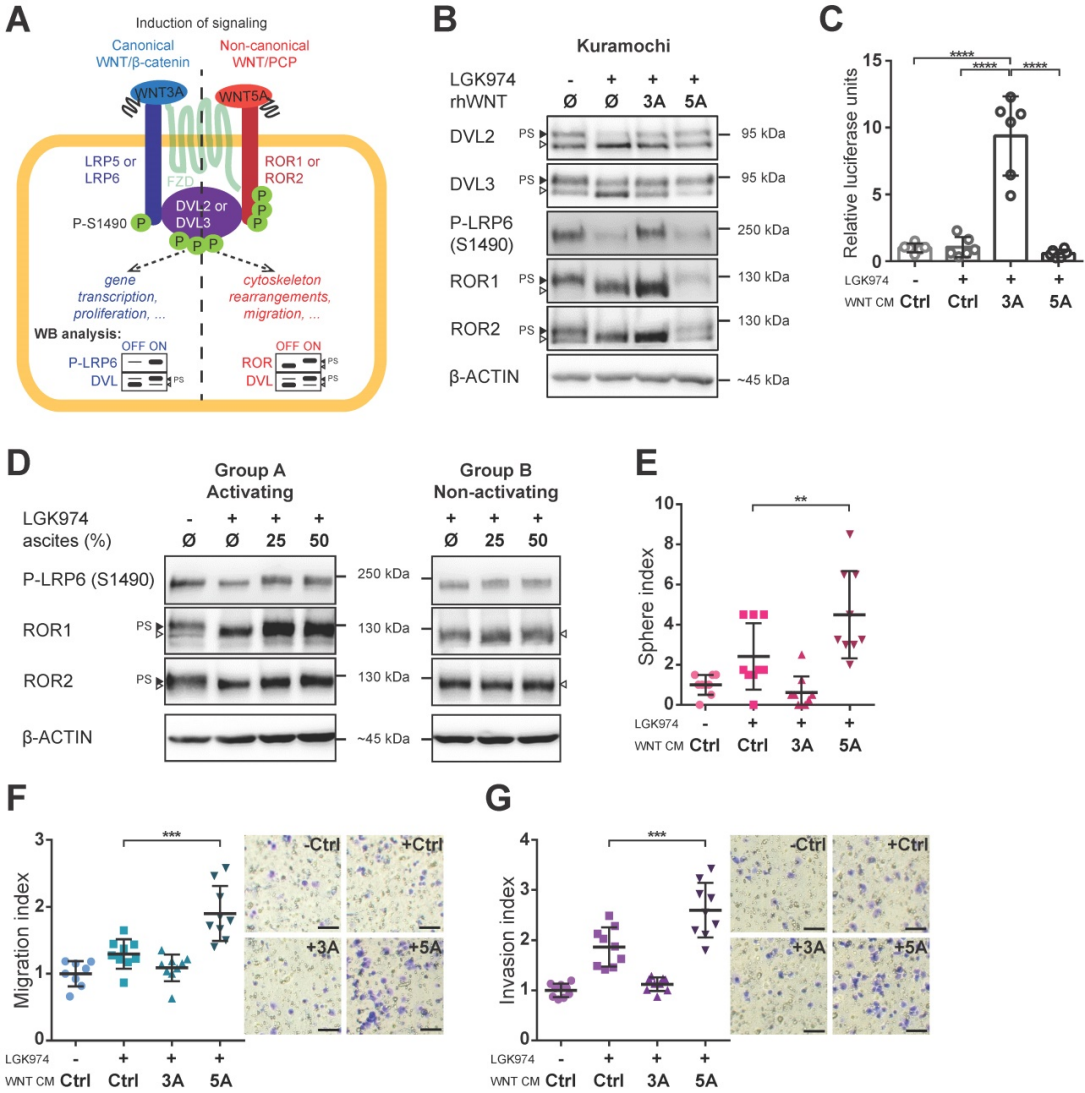
** Both branches of WNT signaling can be induced in HGSC cells, but only WNT/PCP pathway is important for acquisition of metastatic traits. A)** Schematic of the WNT signaling pathways and their outcomes including WB analysis used to assess the activation of WNT canonical (left part, in blue) vs. WNT non-canonical/PCP signaling (right part, in red). Active WNT signaling results in phosphorylation-induced electrophoretic shift of DVL proteins. Phosphorylation of LRP6 on S1490 and electrophoretic shift of ROR receptors, respectively, differentiates between activation of canonical (WNT/β-catenin) by WNT3A and non-canonical (WNT/PCP) signaling by WNT5A. **B)** WNT3A activates WNT/β-catenin signaling, while WNT5A activates WNT/PCP signaling in Kuramochi cell line. **C)** Kuramochi cells are able to activate transcription of TOPFLASH luciferase reporter, which is dependent on full activation of WNT/β-catenin pathway, upon stimulation by WNT3A CM. **D)** Group A ascites activates both canonical and non-canonical WNT signaling, while group B ascites do not activate either of the pathways. **E)** WNT5A (but not WNT3A) increases the self-renewal potential of single cells in sphere forming assay. **F-G)** WNT5A induces migration potential (F) as well as the invasion capacity of HGSC cells in a transwell assay (G). Results are expressed as sphere, migration or invasion index, which is a value normalized to the mean of triplicates of the DMSO control in each experiment. Representative images of transwell membranes are shown on the right side of the corresponding graph, scale bar = 100 μm. Data information: In (C, E-G), data is presented as mean ± SD. ** = *P* ≤ 0.01; *** = *P* ≤ 0.001. **** = *P* < 0.0001 (Tukey post-hoc test of one-way ANOVA). Experiments were done in triplicate, N = 3. Quantification of WBs is available in Supplementary Fig. S1A' and S1B' and S2A-F.

Having identified this tentative clinical correlation between WNT signaling activity in ascites and patients' outcome, we asked which branch of WNT signaling is activated in HGSC. WNT ligands can activate canonical (WNT/β-catenin; prototypical ligand: WNT3A) and non-canonical (β-catenin independent; prototypical ligand: WNT5A) pathways, among which WNT/PCP is the most prominent (reviewed in 7). It is of note that the activation of dedicated membrane co-receptors can be used to dissect WNT pathway activity (Fig. 2A). While phosphorylation of LRP6 on S1490 is specific for WNT/β-catenin signaling, the electrophoretic shift of ROR1 and ROR2 receptors is specific for activation of WNT/PCP signaling. As shown in Fig. 2B, Kuramochi cells responded to both WNT3A and WNT5A and were able to distinctly activate both branches of WNT signaling (Fig. 2B, quantified in Supplementary Fig. S2A-C). Additionally, WNT3A but not WNT5A was able to activate transcription of the luciferase reporter TOPFLASH 29 which confirmed the full competency of Kuramochi cells in the activation of WNT/β-catenin target genes (Fig. 2C, Supplementary Fig. S3A). Importantly, when ascites were assessed for their ability to activate either WNT/β-catenin or WNT/PCP pathway, we found that ascites in group A but not in group B induced both WNT/β-catenin and WNT/PCP pathway (Fig. 2D, Supplementary Fig. S2D-F).

### WNT5A-induced signaling promotes metastatic stem cell-like behaviors of HGSC cells

With a suitable *in vitro* model in hand, we then asked whether the activation of WNT signaling would induce changes in HGSC cells which are known to be associated with cancer progression. HGSC metastasis is the result of multiple intraperitoneal events, whereupon tumor cells are shed from the primary tumor, survive in ascites in the form of spheroids, migrate towards and invade the peritoneal mesothelium. These tumor cells also migrate into the submesothelial matrix to seed and proliferate establishing secondary lesions on the bowel, diaphragm, omentum, and other sites. For this task, the acquisition of key metastatic stem cell (metSC)-like features 30, *i.e.* self-renewal and migration/invasion capacity, is required. Hence, we tested the ability of WNT ligands to induce these behaviors in Kuramochi cells. Interestingly, WNT5A (unlike WNT3A) induced the self-renewal potential of HGSC cells at the single-cell level in the sphere forming assay (Fig. 2E). Similarly, only WNT5A was able to promote chemotactic migration (Fig. 2F) and invasion of HGSC cells as demonstrated in the transwell assay (Fig. 2G). To rule out any possible artefacts created by using WNT CM in our experiments, we also conducted all experiments with recombinant WNT proteins with the results being in parallel with those seen with WNT CM (Supplementary Fig. S3B-E). Similarly, only WNT5A increased chemotactic migration, a measurement that was monitored independently in real-time using the xCELLigence system (Supplementary Fig. S3E, S3F). These results demonstrated that WNT5A (but not WNT3A) can activate a non-canonical WNT pathway that promotes the features - migration, invasion, self-renewal - required for HGSC progression in Kuramochi cells.

### Pharmacologic as well as genetic inhibition of WNT signaling block the effect of WNT5A

**Figure 3:**
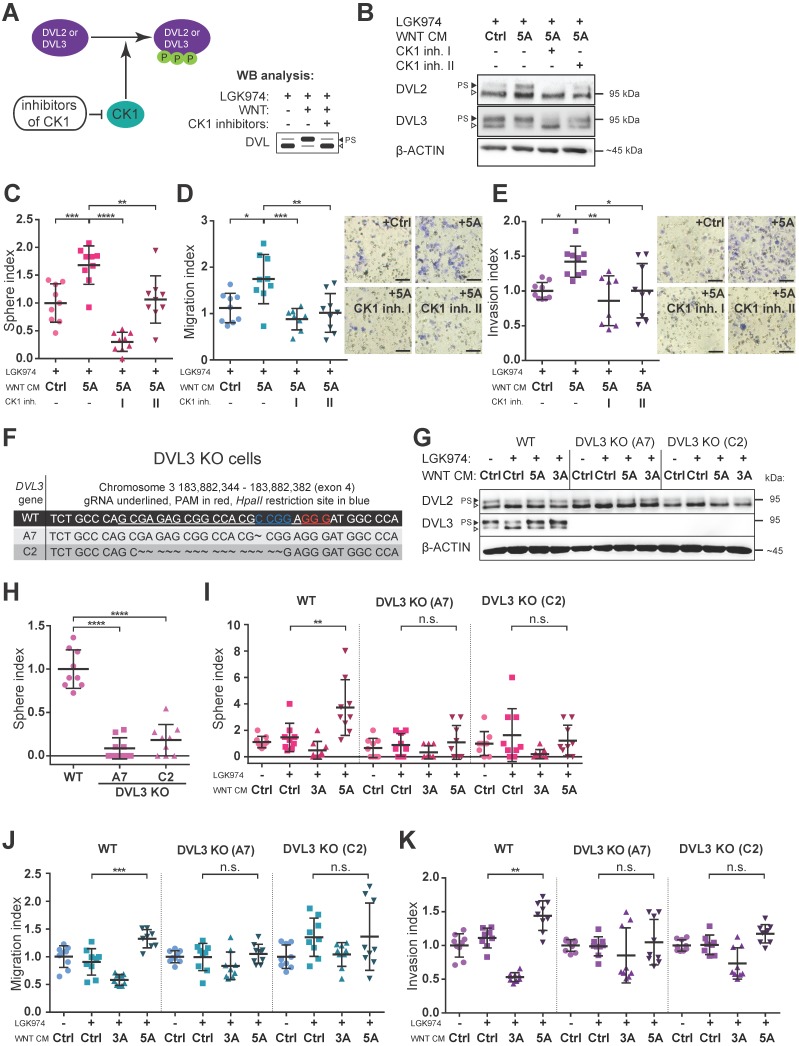
** Inhibition of WNT signaling blocks the effect of WNT5A. A)** Scheme of action and outcomes of CK1 and CK1 inhibitors in the WNT pathway. **B)** Representative WBs from cells used in C-E). Both CK1 inhibitors (CK1 inh. I: 5 μM PF670462, CK1 inh. II: 5 μM D4476) are able to effectively block WNT signaling induced by WNT5A, witnessed by diminishment of PS-DVL2 and PS-DVL3 (black triangle). Quantification of WBs is available in Supplementary Fig. S2G-H. **C-E)** Cells were pretreated with 0.1 μM LGK974 (inhibitor of Porcupine) and then with either control or WNT5A CM for 18 h. The two conditions treated with WNT5A also contained CK1 inhibitors during the whole WNT5A CM treatment. Then the cells were used for functional analyses. **C)** Activation of WNT/PCP pathway by WNT5A increases self-renewal potential of single cells in sphere forming assay. WNT5A induces migration potential (**D**) as well as the invasion capacity of HGSC cells in a transwell assay (**E**) and this effect can be reduced or completely blocked by inhibitors of CK1. Results are expressed as sphere, migration or invasion index, which is a value normalized to mean of the triplicate control condition in each experiment. Representative images of transwell membranes are shown on the right side of corresponding graph, scale bar = 100 μm. **F-K)** Genetic ablation of WNT pathway diminished the effect of WNT5A in functional assays. DVL3, a critical component of WNT pathway, was inactivated in Kuramochi cells using the CRISPR/Cas9 system. **F)** Targeting strategy and sequencing results of individual DVL3 KO clones. Frame shift mutation in exon 4 was introduced into each allele, thus generating homozygous knock-out cell lines. **G)** WB analysis of DVL3 and DVL2 in wild-type (WT) and two DVL3 KO clones (A7 and C2) used in functional assays in H-K, β-ACTIN served as a loading control. **H)** DVL3 KO cells have reduced the ability to form spheres in comparison to WT cells. **I)** WNT5A increased the self-renewal potential of single cells in a sphere forming assay on WT cells but not in DVL3 KO clones. Similarly, WNT5A promotes migration (**J**) and invasion (**K**) in WT cells but not in DVL3 KO cells. Data information: In (C-E, H-K), data is presented as mean ± SD. * = *P* ≤ 0.05; ** = *P* ≤ 0.01; *** = *P* ≤ 0.001. **** = *P* < 0.0001 (Tukey post-hoc test of one-way ANOVA), n.s. = non-significant (*P* > 0.05). Experiments were done in triplicate, N = 3.

Next, we asked whether blocking of WNT/PCP pathway would interfere with WNT5A-induced malignancy-associated self-renewal and migration/invasion capacity of HGSC cells. Casein kinase 1 (CK1) phosphorylates DVL and is an essential regulator in the WNT pathway (Fig. 3A) 24. Inhibition of CK1, particularly of delta and epsilon isoforms, is commonly used to block WNT pathway in experimental settings 31. Treatment of Kuramochi cells with two different inhibitors of CK1 (CK1 inh. I: PF670462; CK1 inh. II: D4476) blocked WNT pathway activation (Fig. 3B, Supplementary Fig. S2G-H) and precluded positive WNT5A effects on sphere forming capacity as well as the inhibition of migration and invasion of these cells (Fig. 3C, 3D, 3E). PF670462 (IC_50_ values of 7.7 nM for CK1ε and 14 nM for CK1δ 32) was more efficient than D4476 (IC_50_ = 300 nM for CK1δ 33) when used at the same concentration in inhibiting WNT5A-induced signaling (evidenced by a shift in DVL) and WNT5A-induced sphere formation and migration/invasion properties of HGSC cells.

To complement data obtained by the pharmacological inhibition of CK1, we turned to the genetic manipulation of Kuramochi cells via the CRISPR/Cas9 system. We generated Kuramochi cells deficient in DVL3 by knocking-out (KO) *DVL3*, a critical component of WNT transduction machinery. Two different DVL3-null Kuramochi cell lines, A7 and C2, (for characterization see Fig. 3F, 3G and Supplementary Fig. S4A) were used in further studies. These DVL3 KO cell lines showed a reduction in their basal sphere formation capacity in comparison to wild-type (WT) Kuramochi cells (Fig. 3H), but had similar basal migration capacity (Supplementary Fig. S4B). The difference in basal sphere forming capacity was not due to the altered proliferation rate, as the DVL3 KO cells proliferated similarly to WT cells. This could be evidenced by the daily enumeration (Supplementary Fig. S4C, S4D, S4D', S4D''), as well as by similar proliferation rates in the long term parallel cell culture of all the cell lines used. Both clones of DVL3 KO Kuramochi cells did not respond to WNT5A in any functional assays - WNT5A failed to promote sphere formation (Fig. 3I), migration in the transwell assay (Fig. 3J) as well as cellular invasion (Fig. 3K) in DVL KO cells although still active in WT cells which were analyzed in the same experiment. In conclusion, both the pharmacological inhibition of WNT signaling (CK1 inhibitors) and genetic deficiency (DVL3 KO) resulted in the loss of WNT5A-induced effects in Kuramochi cells, suggesting that WNT5A indeed induces pro-metastatic behavior in this model via non-canonical WNT5A-CK1-DVL3 axis.

### Patient spheroids isolated from group an ascites are responsive to WNT signaling

Analyses (as depicted in Figs. 1-3) suggest that ascites constitutes, at least in some patients, the environment promoting the metastatic behavior of cancer cells released into the peritoneum. Therefore, we decided to test directly if the patient cancer cells from the WNT pathway activating ascites respond to WNT pathway stimulation. We analyzed the capacity of each ascites to activate the WNT pathway (see Fig. 1) and in parallel also isolated fresh cancer spheroids from the ascites of a few patients. Cancer spheroids which typically show increased chemotherapy and radiation resistance, represent the main form of tumor cells that survive within ascites 34, 35. These multicellular aggregates are believed to be responsible for development of metastasis and recurrent disease.

**Figure 4:**
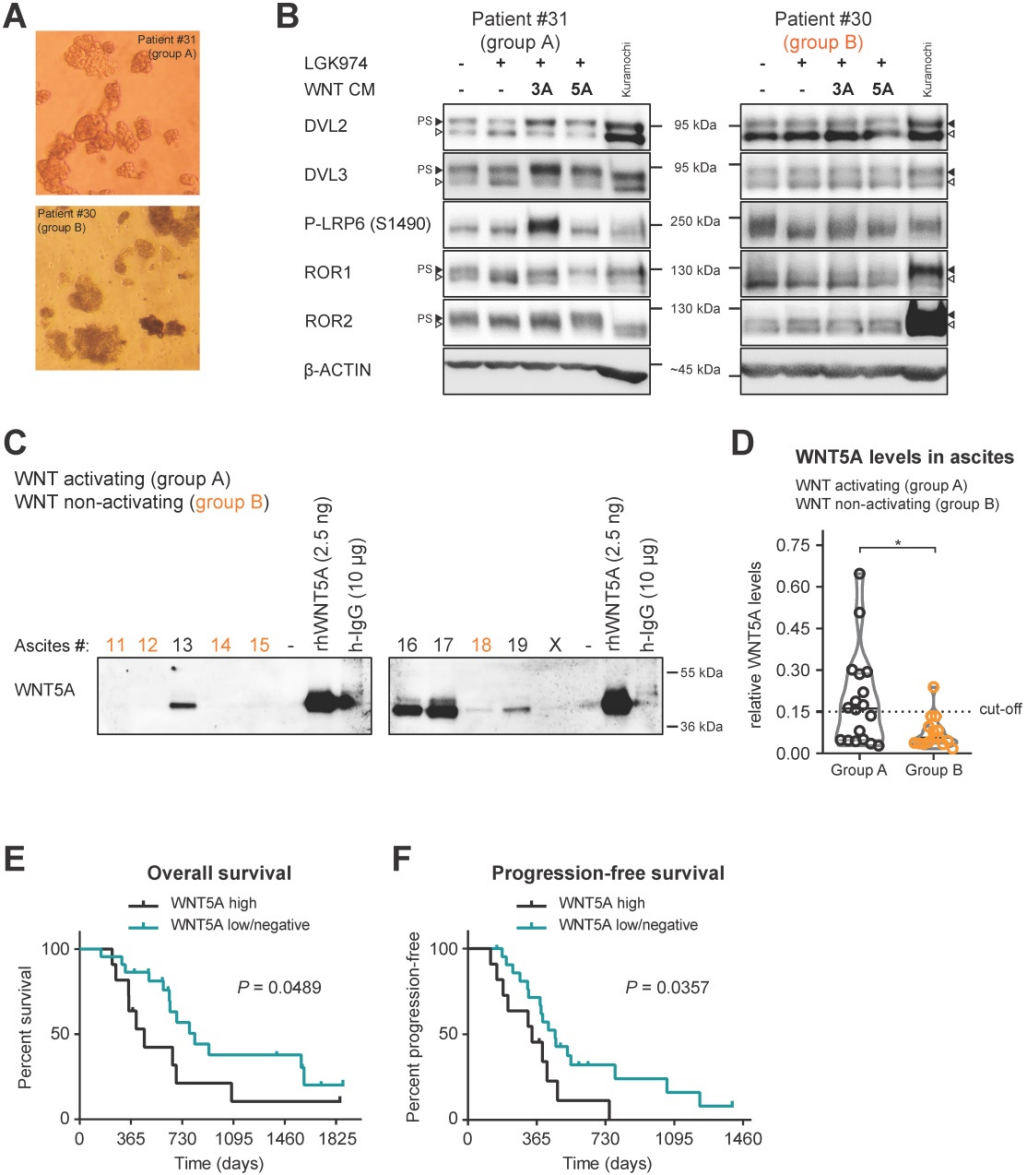
** Patients spheroids are responsive to WNT signaling and WNT5A ligand is present in ascites of group A patients. A)** Representative images of isolated patient spheroids used in WB in B-C. **B)** Left, Group A spheroids have PS-DVL that is reduced upon LGK974 treatment and increased after WNT3A and WNT5A stimuli. This is indicative of endogenous WNT secretion and responsiveness to exogenous WNT ligands similarly as Kuramochi cell line. Right, unchanged PS-DVL levels of group B spheroids indicate that these cells did not secrete endogenous WNTs nor responded to activation by exogenous WNT ligands. **C-D)** WNT5A ligand is present in malignant ascites in group A patients. Non-cellular parts of ascites were fractionized using size exclusion chromatography to remove major serum contaminants such as albumin and immunoglobulins (IgG). SDS-PAGE gels were immunoblotted with WNT5A antibody. The fraction containing the majority of WNT proteins is shown for each ascites. Recombinant WNT proteins are shown as controls and indicators of correct molecular weight (MW), unlike human IgG which has a similar MW which can be mistaken for WNTs if not depleted (false positive signal from secondary antibody). Group B ascites are marked by orange color. Representative image is shown. X - patients excluded from the study. WBs from all ascites were simultaneously processed as seen in Supplementary Fig. S5A, and quantified in S5B and **D)** Violin plot. Group A ascites contain higher levels of WNT5A protein than Group B ascites. *P* = 0.0186, Student´s t-test. 0.15 was set as a cut-off value for the analysis of the patient outcome. **E)** OS of patients with WNT5A high and WNT5A low/negative ascites. **F)** PFS of patients with WNT5A high and WNT5A low/negative ascites.

Patient spheroids isolated from ascites (Fig. 4A), that could activate WNT signaling (group A), were hyperphosphorylated at DVL2 and DVL3 when analyzed by WB. PS-DVL could be reverted by LGK974 treatment blocking their autocrine WNT production (Fig. 4B, left panel), which is indicative of endogenous WNT secretion. These cells also retain responsiveness to exogenous WNT3A and WNT5A similarly to Kuramochi cell line (Fig. 4B, left panel). Importantly, cancer spheroids isolated from group B ascites, *i.e.* non-activating WNT signaling, showed limited phosphorylation of DVL2 and DVL3 that was not further reduced by LGK974 treatment (Fig. 4B, right panel). This suggests that these spheroids do not produce autocrine WNTs; furthermore, they did not respond to exogenous WNT stimulation. This conclusion is also supported by phosphorylation of the co-receptors LRP6, ROR1 and ROR2 that showed ligand-dependent activation only in spheroids from group A patients. Together, this analysis confirmed that WNT-inducing activity in patient ascites triggers the response in the present cancer spheroids and that ascites act as a milieu that mediates the long distance action of this signaling activity.

### Ascites capable of activating WNT signaling contains several WNT ligands

Our initial analysis showed that in a significant portion of patients (55%, group A) ascites could activate WNT signaling. Importantly, we showed that the WNT/PCP pathway, rather than the WNT/β-catenin pathway, was functionally important for the acquisition of metastatic behaviors of HGSC cells. Thus, we investigated what is the source of non-canonical WNT-inducing activity in the ascites. Ascites samples are rich in serum proteins that have similar molecular weight as WNT ligands (mainly albumin and immunoglobulins), making detection of WNT ligands in whole ascites samples by WB challenging. Therefore, we performed fractionation of ascites using size exclusion chromatography and focused on the detection of the prototypic non-canonical WNT ligand WNT5A in patient ascites by WB. As shown in Fig. 4C, patients from group A have detectable amounts of WNT5A protein in their ascites in comparison to group B patients. The quantification of WNT5A levels in all analyzed ascites (Fig. 4D, raw data in Supplementary Fig. S5) showed that WNT5A levels were negligible in most ascites from group B which were incapable of WNT induction in the recipient cells. When ascitic samples were divided into WNT5A high (N=11) and WNT5A low/negative (N=22) (see Fig. 4D), patients with high levels of WNT5A protein in their ascites had worse OS (Fig. 4E) and PFS (Fig. 4F). This correlation suggests that WNT-activating factors in the ascites include WNT5A, and in a broader view, supports the existence of a positive prometastatic feedback loop that is driven by WNT ligands within ascites.

### WNT/PCP components are rarely mutated in HGSC

Finally, we asked whether HGSC tumors harbor molecular alterations in the WNT/PCP pathway that could explain their sensitivity towards WNT5A. The WNT/PCP pathway is evolutionary conserved from *Drosophila* to humans and is crucial for the establishment of cell polarity. In general, WNT/PCP components interact with each other across the cell membrane and intracellularly to sequester two protein complexes from opposite sides of the cell providing the polarity in plane for epithelial cells and orientation for the polarized behavior of mesenchymal cells 8. We selected a panel of 30 WNT/PCP genes for detailed analysis (Supplementary Table ST2). These gene families encode for core PCP components (CELSR, FZD, DVL, ANKRD, VANGL and PRICKLE) as well as for vertebrate specific PCP-associated factors (WNT, CTHRC1, ROR, RYK, PTK7) 8. We did not include the effectors (small GTPases, *etc.*) as part of our analysis, as these may be largely affected by inputs from other signaling pathways. We performed *in silico* analysis of the WNT/PCP genes DNA alterations in HGSC using the COSMIC database 36. First, we found that the WNT/PCP genes are rarely mutated in HGSC, CELSR3 mutations were most recurrent and were found in 0.74% of samples. On the contrary, no mutations were identified in *CSNK1A1, CSNK1D, DVL1, DVL2, DVL3, FZD2, FZD6, FZD10, PRICKLE3, VANGL1,* and *WNT5A* (N=673-693) (Supplementary Table ST2).

### WNT/PCP components are differentially expressed during disease progression and their expression levels are independent prognostic factors for overall survival in TCGA cohort

**Figure 5:**
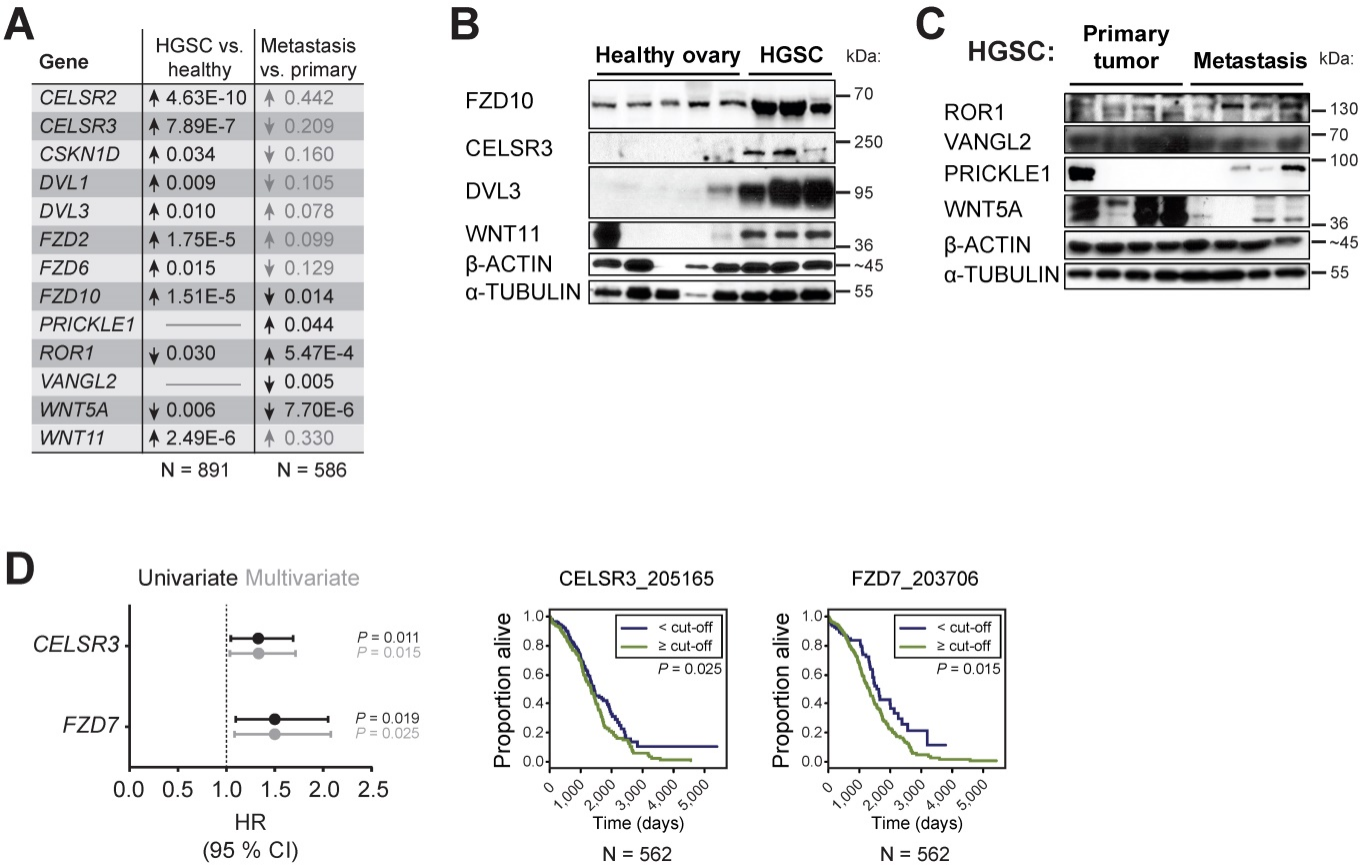
** WNT/PCP components are differentially expressed during HGSC progression. A)** WNT/PCP components are differentially expressed in HGSC. Summary of meta-analysis of datasets from Oncomine database, *P* values > 0.05 are in grey. Detailed information and whole analysis is provided in Supplementary Fig. S6B-C. **B-C)** Verification of the Oncomine transcriptomics results on primary patient samples on the protein level by WB. WNT/PCP components (FZD10, CELSR3, DVL3 and WNT11) are overexpressed in HGSC in comparison to healthy ovarian tissue (**B**). **C)** VANGL2 and WNT5A levels were decreased in metastatic tissue in comparison to primary site, while ROR1 and PRICKLE1 levels were upregulated. Quantification of WBs is available in Supplementary Fig. S7. **D)** Univariate and multivariate results of Cox proportional hazard models and Kaplan-Meier curves of overall survival in patients of TCGA Ovarian dataset for genes upregulated in HGSC. Detailed analysis is provided in Supplementary Table ST3.

Next, we focused on the transcriptome and analyzed the microarray data available through the Oncomine database 37. A subset of WNT/PCP genes is overexpressed in OC in comparison to other types of cancer indicating a specific role for this pathway in OC progression. This subset includes *FZD2, FZD6, PTK7, RYK, CELSR1, CELSR2, VANGL2, ANKRD6,* and *DVL3*, which were overexpressed across 15 available studies for meta-analysis (Supplementary Fig. S6A). A meta-analysis of HGSC versus healthy ovary revealed the consistent overexpression of 10 genes: *WNT11, FZD2, FZD6, FZD10, CELSR2, CELSR3, CSKN1D, DVL1*, *DVL3,* and *PRICKLE3* (Fig. 5A and Supplementary Fig. S6B). Using our collection of HGSC patient samples and validated antibodies, we were able to prove overexpression of WNT11, FZD10, CELSR3 and DVL3 genes in HGSC on protein level (Fig. 5B, Supplementary Fig. S7A). Additionally, a meta-analysis of metastasis versus primary site samples in HGSC revealed overexpression of 6 genes: *CTHRC1, FZD7, ROR1, ROR2, VANGL1* and *PRICKLE1* and underexpression of 3 genes: *WNT5A, FZD10,* and* VANGL2* (Fig. 5A, Supplementary Fig. S6C). Using our collection of HGSC patient samples, we were able to show that significant changes observed at the mRNA level for *ROR1, PRICKLE1, WNT5A, and VANGL2* (Fig. 5A) are translated into changes at the level of protein in metastasis vs. primary site (Fig. 5C, Supplementary Fig. S7B). Finally, a multivariate analysis of progression in more than 550 patients with HGSC included in The Cancer Genome Atlas (TCGA) dataset 16 showed that low expression of *FZD7* and *CELSR3* was favorable to overall patient survival (Fig. 5D, Supplementary Table ST3). Altogether, this meta-analysis backed up by protein analysis of our samples pointed to clinical relevance of alterations in the expression profile of WNT/PCP pathway components and further strengthened the link between the non-canonical WNT pathway and progression of HGSC.

## Discussion

Our limited knowledge of the molecular mechanisms associated with HGSC progression results in the inability to reverse the overall poor prognosis of HGSC. Malignant ascites are a typical feature of HGSC that occurs more commonly in HGSC than in any other tumor type 38. Ascites are known to promote transcoelomic spread of the disease resulting in the intraperitoneal metastases. In agreement with the generally procancerous nature of ascites, it has been shown that soluble factors contained in ascites can provide extracellular cues that drive cancer progression via increased cell renewal, proliferation, migration, and invasion 35, (also reviewed in 39). Our study adds the WNT ligand WNT5A to the list of prometastatic soluble factors present in the ascites of HGSC patients. We propose that WNT5A is an important component of ascites, the tumor microenvironment that in turn helps to spread these pro-metastatic factors. Subsequently, patients with high levels of WNT5A protein in their ascites present a worse course of disease than patients with low levels. It is tempting to speculate what may be the source of WNT ligands in malignant ascites. Unfortunately, our study does not provide a definitive answer but points to cancer cells themselves as the first candidates. We have demonstrated that both Kuramochi cells as well as primary patient spheroids secrete WNTs and are capable of producing an autocrine signaling loop. Tumor associated macrophages (TAMs) are additional candidates for the source of WNTs, as they are known to produce WNT5A in ovarian cancer 35 and in multiple other contexts 40.

Our study defines the functional importance of the non-canonical WNT5A-driven pathway in the progression of HGSC. We have shown that the activation of WNT/PCP signaling endowed HGSC cells with increased self-renewal and the ability to consistently migrate/invade and that the inhibitors of WNT pathway block these prometastatic features. We demonstrated that a portion of the patients who lack this positive feedback loop in their tumor microenvironment present with less aggressive disease and have a significantly better outcome. This is despite the fact that patients with ascites at the time of surgery already represent a cohort with a poor prognosis 41. However, the data is based on a limited number of patients and should be tested on more extensive cohorts.

Canonical WNT signaling is known to play a critical role in (cancer) stem cell maintenance 7 and metastasis, including ovarian cancer and breast cancer, as confirmed by recent studies 42-44. On contrary, we found that only treatment with WNT5A increased sphere formation capacity, a surrogate marker for stem cell-like properties. Furthermore, WNT5A stimulation resulted in the increased chemotactic migration and invasion potential of HGSC cells, which is consistent with the pro-migratory role of WNT/PCP pathway 8. The effect of WNT5A was efficiently blocked using genetic ablation of critical WNT component DVL3 but also by the pharmacological inhibition of CK1, a DVL activating enzyme. It remains to be seen if CK1-mediated processes represent some type of vulnerability in HGSC that could be pharmacologically targeted. It is of note, although selective CK1 inhibitors have not yet reached clinical trials, compounds such as PF670462 have been shown to be beneficial and are well-tolerated *in vivo* preclinical mouse studies 31, and thus could be used in a wide variety of WNT-driven cancers, including breast 45 and ovarian cancer (this study and 18).

We have demonstrated that ascites in many patients are capable of inducing WNT signaling in tumor cells and also shown that tumors exhibit alterations in the expression level of WNT/PCP components which may explain their sensitivity to WNT5A. In the functional tests that we conducted, we used Kuramochi cells which harbor a molecular profile closest to primary HGSC cells 17. It is noteworthy to remind the reader that the OC research community has worked for decades with “traditional HGSC” cell lines which according to a recent study 17 poorly represent the genomic features of a majority of OC patients. Thus, despite the numerous studies in the literature on WNT components in OC (summarized in 6), there is only a limited relevance because of using cell lines which are quite distant from real HGSC patient samples.

The fact that patient ascites were able to induce either both WNT pathway branches and none indicated that both WNT/β-catenin and WNT/PCP signaling may play an important role in HGSC progression. In addition to the separate functions of WNT pathways, there is strong evidence that the WNT pathways can also influence, mostly antagonize, each other 46, 47. Which WNT pathway will be ultimately activated is dependent on the receptor/ligand combination, rather than on specific WNT ligands 48. Both Kuramochi and patient HGSC cells express both types of receptors and consequently are able to activate both β-catenin dependent and independent signaling after stimulation with respective ligands. This is in agreement with a transcriptome-based analysis of cells isolated from ascites which also reported expression of both-canonical and non-canonical ligands on ovarian cancer cells and TAMs 35.

In order to dissect the potential receptor-ligand combination(s) that mediate the prometastatic action of WNT pathway in HGSC, we performed *in silico* meta-analysis of available studies that describe the mutational landscape and transcriptome of HGSC. WNT/PCP genes were not mutated in HGSC but expression of several WNT/PCP genes was greatly upregulated in OC (tumor vs. normal tissue) in comparison to other tumors types, stressing the importance of the pathway for progression of OC. In line with previous reports 49, the expression of WNT5A was downregulated in HGSC in comparison to healthy ovarian tissue and in metastasis vs. primary tumor site (also confirmed on the protein level by WB). Some researchers 50-52 have reported WNT5A upregulation in all major subtypes (serous, mucinous, endometrioid and clear cell) of epithelial ovarian cancer (EOC) and its association with a worse prognosis 50, 51. Our meta-analysis of TCGA dataset, however, did not find a significant association with OS by multivariate Cox proportional hazards analysis. This data underlies the pleotropic effects of WNT5A seen in the development of different types of female cancers 53. FZD2 was shown to correlate with poor survival of HGSC patients and represents a candidate receptor that can mediate WNT5A effects in OC 54. On the other hand, WNT11 was upregulated in HGSC, which is in line with a previous report showing its expression on mRNA level of cancer cells which were isolated from ascites 35. Concomitantly, receptor tyrosine kinase-like orphan receptors (RORs), which are the major receptors for WNT5A (as well as other “non-canonical” WNT ligands such as WNT11) were downregulated in HGSC but upregulated in metastases vs. primary sites. These findings are in direct contrast to previous reports that RORs are upregulated in EOC 20, 55. However, these studies investigated EOC as one disease, while we assessed only HGSC, the most lethal type of EOC. The significance of ROR receptors in HGSC was supported further by studies that showed that ROR1 is overexpressed in ovarian cancer stem cells (CSCs) and can be effectively targeted for anti-cancer-stem-cell therapy 56. Finally, the lower expression of FZD7 correlated with better OS of the patients and was upregulated in metastases which is in agreement with FZD7 overexpression in Stem-A subtype of HGSC. *In vitro*, FZD7 drove the aggressiveness of this particular HGSC subtype by regulating cell proliferation, cell cycle progression, maintenance of the mesenchymal phenotype, and cell migration capability via casein kinase 1ε (CK1ε)-mediated WNT/PCP pathway 57. This and other studies 18, 20-22, 58 in the context of our findings point toward the crucial role of WNT5A/ROR/DVL/CK1 axis in the progression of HGSC.

In conclusion, using the functional test of WNT signaling inducing capacity (or its surrogate marker - WNT5A protein levels) of ascites, we have identified a subgroup of HGSC patients whose prognosis is better than could be judged from other independent prognostic factors. Although ascites were able to induce both canonical and non-canonical WNT signaling, only the non-canonical branch (WNT/PCP pathway) was relevant for acquisitions of metastatic traits by functional assays. Our approach allowed us to pinpoint the role of the WNT/ROR/DVL/CK1 axis in the progression of HGSC as well as the ability to bridge the gap between published observations made on clinical samples and the molecular mechanisms revealed in less appropriate HGSC cell lines models. Our data serves as the basis for further experimental directions with possible therapeutic and/or translational potential.

## Supplementary Material

Supplementary figures and tables.

## Abbreviations

ACK: ammonium-chloride-potassium
ANKRD6: ankyrin repeat domain 6
ANOVA: analysis of variance
BSA: bovine serum albumin
CELSR: cadherin EGF LAG seven-pass G-type receptor
CFSE: Carboxyfluorescein succinimidyl ester
CK1: casein kinase 1
CM: conditioned medium
COSMIC: Catalogue of Somatic Mutations in Cancer
CRISPR: Clustered Regularly Interspaced Short Palindromic Repeats
CST: Cell Signaling Technology
CTHRC1: collagen triple helix repeat containing 1
Ctrl: control
MCDB/DMEM: MCDB/Dulbecco Modified Eagle's Medium
DMSO: dimethyl sulfoxide
DVL: disheveled
EOC: epithelial ovarian cancer
FBS: fetal bovine serum
FIGO: the International Federation of Gynecology and Obstetrics
FZD: frizzled
HGSC: high grade serous carcinoma of the ovary, fallopian tube and peritoneum
KO: knock-out
LRP: lipoprotein receptor-related
metSC: metastatic stem-cell
OC: ovarian cancer
OS: overall survival
PBS: phosphate buffered saline
PCP: planar cell polarity
PCR: polymerase chain reaction
PFS: progression-free survival
PS: phosphorylated and shifted
PTK7: protein tyrosine kinase 7
rh: recombinant human
ROR: receptor tyrosine kinase-like orphan receptor
RPMI: Roswell Park Memorial Institute
RTCA: Real Time Cell Analysis
RYK: receptor-like tyrosine kinase
SCBT: Santa Cruz Biotechnology
SDS-PAGE: sodium dodecyl sulfate-polyacrylamide gel electrophoresis
STR: short tandem repeat
TAMs: Tumor associated macrophages
TCGA: the Cancer Genome Atlas
VANGL: van Gogh-like
WB: western blotting/blot
WNT: wingless/int-1
