# Supplementary material for: WNT signaling inducing activity in ascites predicts poor outcome in ovarian cancer
Source: Theranostics. 2020 Jan 1;10(2):537–52. doi: 10.7150/thno.37423 (PMC6929979; doi:10.7150/thno.37423)
Supplement: Supplementary file 1 — Supplementary figures and tables. [file thnov10p0537s1.pdf]

SUPPLEMENTARY FIGURE LEGENDS

Supplementary Figure S1: Differential potential to induce WNT signaling separates two groups of ascites/patients.
Quantification of WB results from Fig. 1B, 1D and 2B. Activity of WNT signaling is expressed as the ratio of PS-DVL to total levels of DVL protein. **A)** DVL2, WNT CM. **A')** DVL2, rhWNT. **B)** DVL3, WNT CM. **B')** DVL3, rhWNT. **C-D)** Patients in group A (grey) have an increased ratio (P value < 0.05) of PS-DVL/total DVL in comparison to control (LGK974 treated cells, open bar). Group B patients are shown in orange. Data is presented as mean ± SD. * = P ≤ 0.05; ** = P ≤ 0.01; *** = P ≤ 0.001. **** = P < 0.0001, n.s. = non-significant (P > 0.05). Tukey post-hoc test of one-way ANOVA was used in A-B, paired t-test was used in C-D.

Supplementary Figure S2: Quantification of WB results from Fig. 2B, 2D and 3B.
A) pLRP6, WNT CM. **A')** pLRP6, rhWNT. **B)** ROR1, WNT CM. **B')** ROR1, rhWNT. **C)** ROR2, WNT CM. **C')** ROR2, rhWNT. **D)** pLRP6, ascites. **E)** ROR1, ascites. **F)** ROR2, ascites. **G)** DVL2, WNT5a CM. CKI inhibitor. **H)** DVL3, WNT5a CM. CKI inhibitor. Data is presented as mean ± SD. * = P ≤ 0.05; ** = P ≤ 0.01; *** = P ≤ 0.001. **** = P < 0.0001, n.s. = non-significant (P > 0.05). Tukey post-hoc test of one-way ANOVA was used in A-C, G-H, paired t-test was used in D-F.

Supplementary Figure S3: Functional assays with WNTs.
Kuramochi cells were treated and assays performed exactly as in experiments in Fig. 2 but WNT ligands were provided in the form of recombinant human (rh) proteins. **A)** TOPFLASH assay. **B)** Sphere assay. **C)** Sphere assay in which WNTs and LGK974 were added during the assay to cells pretreated with LGK974. **D)** Migration assay. **E)** Invasion assay. **F-G)** Migration in xCELLigence system, cells were treated with WNT CM (**F**) or rhWNT (**G**). Two time points (4 h and 16 h) are plotted on the right. Data is presented as mean ± SD. * = P ≤ 0.05; ** = P ≤ 0.01; *** = P ≤ 0.001. **** = P < 0.0001 (Tukey post-hoc test of one-way ANOVA), n.s. = non-significant (P > 0.05).

Supplementary Figure S4: Evaluation of DVL3 KO Kuramochi cells.
A) PCR strategy used to select DVL3 KO clones. The amplified fragment was digested with *Hpa*II restriction endonuclease. The *Hpa*II site was disrupted in the mutant allele, which shows only one larger band (349 bp) as opposed to the WT allele which yielded two small bands (185 bp and 164 bp). DVL3 KO cells harbor the same basal migration potential (**B**) and proliferate similarly in basal conditions (**C**) as WT cells. There was also no significant difference in the proliferation rate upon stimulation with WNT CM between WT cells (**D**) and DVL3 KO clones A7 (**D'**) and C2 (**D''**). n.s. = non-significant (P > 0.05) in Tukey post-hoc test of one-way ANOVA.

Supplementary Figure S5: Quantification of WNT5A protein levels in all ascites.
A) The raw data of all ascitic samples were processed simultaneously (as described in Fig. 4C) to allow reliable quantification. X – patients excluded from the study. **B)** Quantification of data from A. 0.15 was set as a cut-off value for segregation into WNT5A high and WNT5A low/negative groups for analysis of patient outcome.

Supplementary Figure S6: Core WNT/PCP components are differentially expressed in HGSC.

The Oncomine™ Platform (Thermo Fisher, Ann Arbor, MI) was used for analysis and visualization. **A)** WNT/PCP genes are overexpressed in OC in comparison to other cancers. A meta-analysis of 15 datasets/studies was performed. It harbors all datasets available containing ovarian cancer data and includes both multi-cancer studies using primary samples (4 in total) as well as 11 studies on cell lines. The respective published studies are indicated in the reference list [1-10]. Genes above the dashed line are significantly overexpressed in OC across the studies (*P* value < 0.05). **B)** WNT/PCP genes are differentially expressed in HGSC in comparison to healthy ovaries. A meta-analysis of datasets/studies was performed. It consists of all datasets available that specifically contain HGSC and healthy ovarian tissue data. The respective studies are indicated in the reference list [11-15]. Genes above the dashed line are significantly overexpressed (red color, left panel) or underexpressed (blue color, right panel) in HGSC over normal ovarian tissue across the studies (*P* value < 0.05). **C)** WNT/PCP genes are differentially expressed in metastasis in comparison with primary tumor. A meta-analysis of all four available studies was performed. The respective studies are indicated in the reference list [11, 16]. Genes above the dashed line are significantly overexpressed (red color, left panel) or underexpressed (blue color, right panel) in secondary over primary tumors across the studies (*P* value < 0.05). **D)** Datasets used in Oncomine analysis. Complete information about datasets is available in Supplementary Table ST4.

Supplementary Figure S7: Quantification of WBs from Fig. 4B and 4C.

Violin plot. * = $P \leq 0.05$; ** = $P \leq 0.01$; *** = $P \leq 0.001$ (unpaired t-test).

Supplementary Table Legends

Supplementary Table ST1: Clinicopathological data of patient cohorts.
Data from a cohort of 33 patients. From April 2013 to July 2018, patients undergoing surgical treatment for OC who were suitable for ascites collection at the Department of Obstetrics and Gynecology, University Hospital Brno were consecutively enrolled into the study.

Supplementary Table ST2: The core WNT/PCP genes are very rarely mutated in HGSC.
Mutational status of the WNT/PCP genes was examined using the COSMIC database. The frequency of mutations in HGSC resulting in change in amino acids sequence was calculated for each gene. Asterisks indicate genes, which were found to be mutated in study by Choi [17]; * indicates patient OSC3, where mutations were found in nine multiregional biopsies of metastatic HGSC, ** indicates patient OSC2, where mutations were found in seven multiregional biopsies of metastatic HGSC. Genes are ordered according to their location within the WNT/PCP pathway and alphabetically within each group which is indicated in the first column.

Supplementary Table ST3: Univariate and multivariate Cox proportional hazard models of overall survival of patients with HGSC in TCGA cohort.
Detailed analysis of individual gene probes retrieved from Oncomine database (TCGA Ovarian dataset). HR with 95% confidence interval represents ratios for OS of patients in the group above the cut-off compared to OS in below the cut-off group. *P* value in bold indicates statistical significance (*P* value < 0.05).

Supplementary Table ST4: Dataset references for Supplementary Fig. S6.

References for Supplementary figures and tables

1. Barretina J, Caponigro G, Stransky N, Venkatesan K, Margolin AA, Kim S, et al. The Cancer Cell Line Encyclopedia enables predictive modelling of anticancer drug sensitivity. Nature. 2012; 483: 603-7.
2. Garnett MJ, Edelman EJ, Heidorn SJ, Greenman CD, Dastur A, Lau KW, et al. Systematic identification of genomic markers of drug sensitivity in cancer cells. Nature. 2012; 483: 570-5.
3. Gyorffy B, Surowiak P, Kiesslich O, Denkert C, Schafer R, Dietel M, et al. Gene expression profiling of 30 cancer cell lines predicts resistance towards 11 anticancer drugs at clinically achieved concentrations. Int J Cancer. 2006; 118: 1699-712.
4. Lee JK, Havaleshko DM, Cho H, Weinstein JN, Kaldjian EP, Karpovich J, et al. A strategy for predicting the chemosensitivity of human cancers and its application to drug discovery. Proc Natl Acad Sci U S A. 2007; 104: 13086-91.
5. Ramaswamy S, Tamayo P, Rifkin R, Mukherjee S, Yeang CH, Angelo M, et al. Multiclass cancer diagnosis using tumor gene expression signatures. Proc Natl Acad Sci U S A. 2001; 98: 15149-54.
6. Ramaswamy S, Ross KN, Lander ES, Golub TR. A molecular signature of metastasis in primary solid tumors. Nat Genet. 2003; 33: 49-54.
7. Scherf U, Ross DT, Waltham M, Smith LH, Lee JK, Tanabe L, et al. A gene expression database for the molecular pharmacology of cancer. Nat Genet. 2000; 24: 236-44.
8. Shankavaram UT, Reinhold WC, Nishizuka S, Major S, Morita D, Chary KK, et al. Transcript and protein expression profiles of the NCI-60 cancer cell panel: an integromic microarray study. Mol Cancer Ther. 2007; 6: 820-32.
9. Staunton JE, Slonim DK, Coller HA, Tamayo P, Angelo MJ, Park J, et al. Chemosensitivity prediction by transcriptional profiling. Proc Natl Acad Sci U S A. 2001; 98: 10787-92.
10. Su AI, Welsh JB, Sapinoso LM, Kern SG, Dimitrov P, Lapp H, et al. Molecular classification of human carcinomas by use of gene expression signatures. Cancer Res. 2001; 61: 7388-93.
11. Adib TR, Henderson S, Perrett C, Hewitt D, Bourmpoulia D, Ledermann J, et al. Predicting biomarkers for ovarian cancer using gene-expression microarrays. Br J Cancer. 2004; 90: 686-92.
12. Bonome T, Levine DA, Shih J, Randonovich M, Pise-Masison CA, Bogomolniy F, et al. A gene signature predicting for survival in suboptimally debulked patients with ovarian cancer. Cancer Res. 2008; 68: 5478-86.
13. Hendrix ND, Wu R, Kuick R, Schwartz DR, Fearon ER, Cho KR. Fibroblast growth factor 9 has oncogenic activity and is a downstream target of Wnt signaling in ovarian endometrioid adenocarcinomas. Cancer Res. 2006; 66: 1354-62.
14. Lu KH, Patterson AP, Wang L, Marquez RT, Atkinson EN, Baggerly KA, et al. Selection of potential markers for epithelial ovarian cancer with gene expression arrays and recursive descent partition analysis. Clin Cancer Res. 2004; 10: 3291-300.
15. Cancer Genome Atlas Research N. Integrated genomic analyses of ovarian carcinoma. Nature. 2011; 474: 609-15.
16. Anglesio MS, Arnold JM, George J, Tinker AV, Tothill R, Waddell N, et al. Mutation of ERBB2 provides a novel alternative mechanism for the ubiquitous activation of RAS-MAPK in ovarian serous low malignant potential tumors. Mol Cancer Res. 2008; 6: 1678-90.
17. Choi YJ, Rhee JK, Hur SY, Kim MS, Lee SH, Chung YJ, et al. Intraindividual genomic heterogeneity of high-grade serous carcinoma of the ovary and clinical utility of ascitic cancer cells for mutation profiling. J Pathol. 2017; 241: 57-66.

Supplementary Figure S1

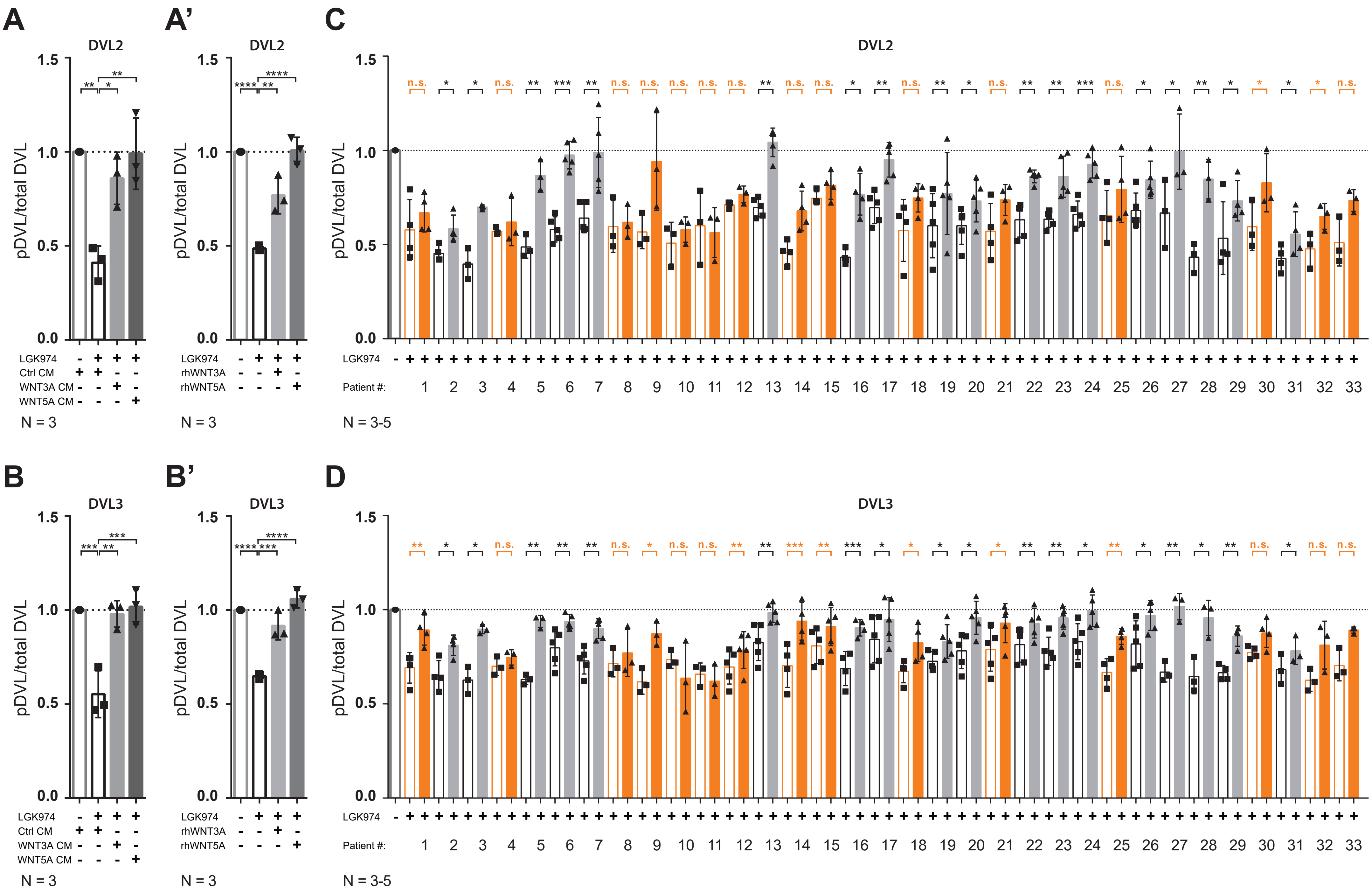

Supplementary Figure S2

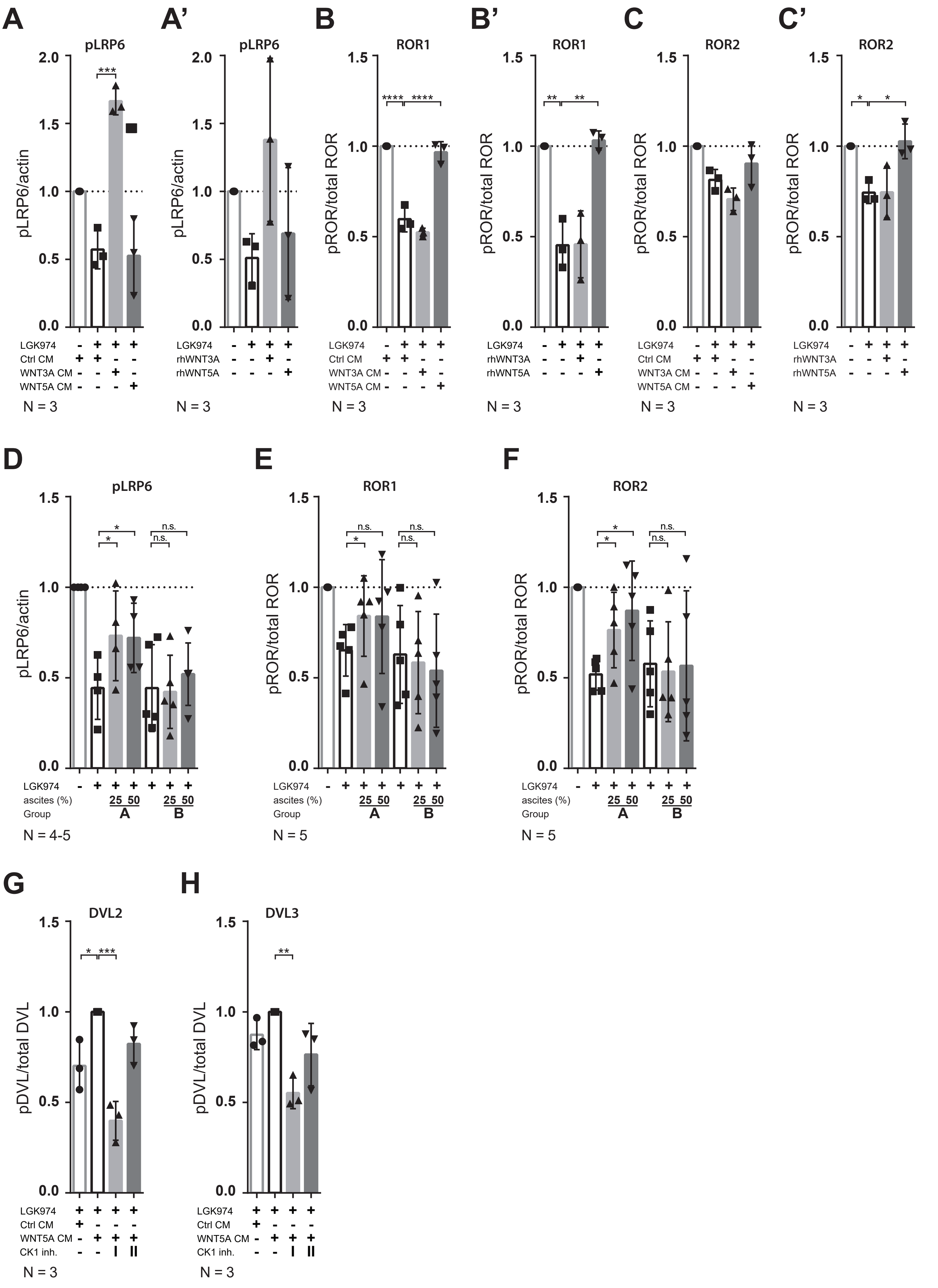

Supplementary Figure S3

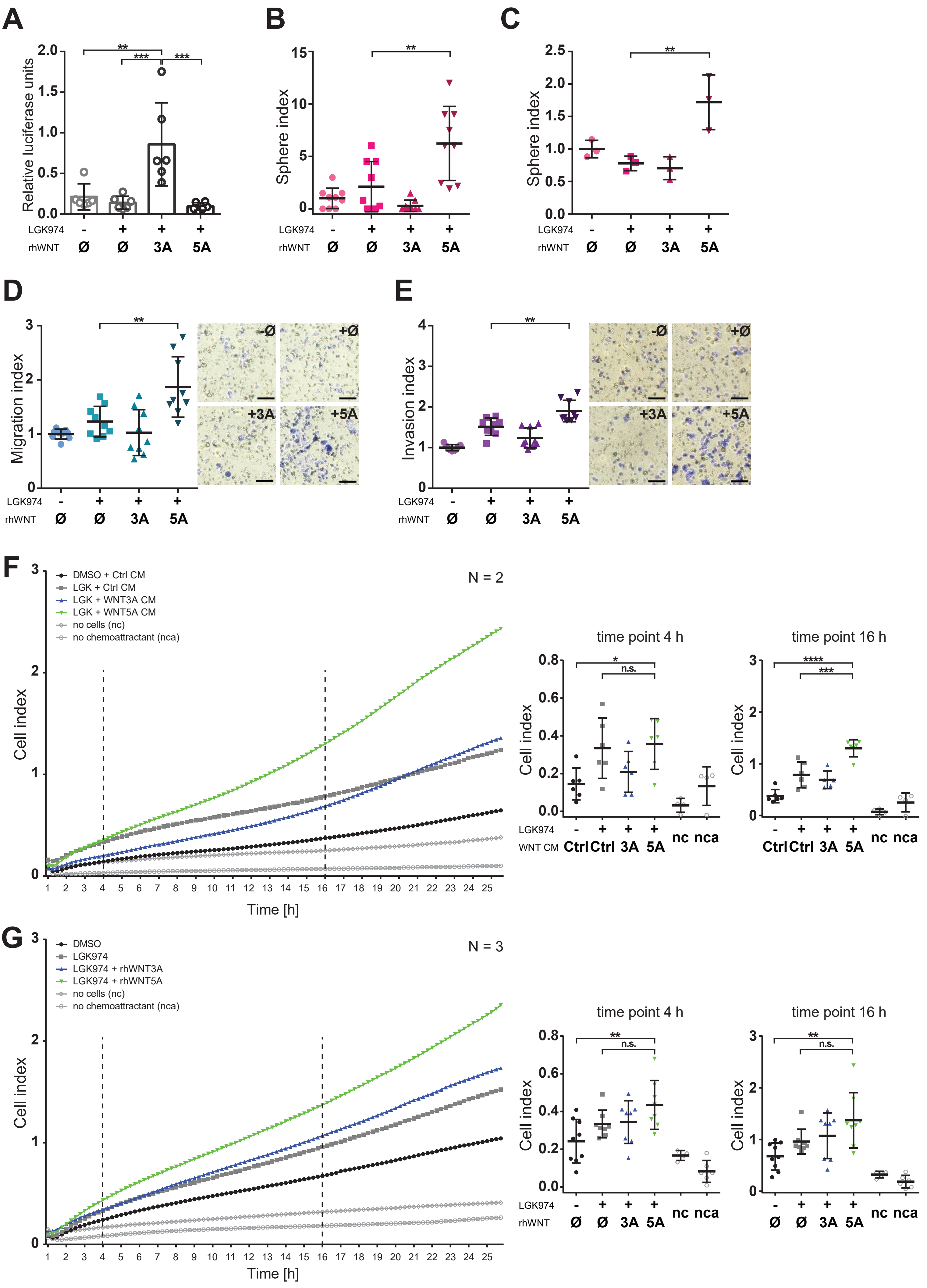

Supplementary Figure S4

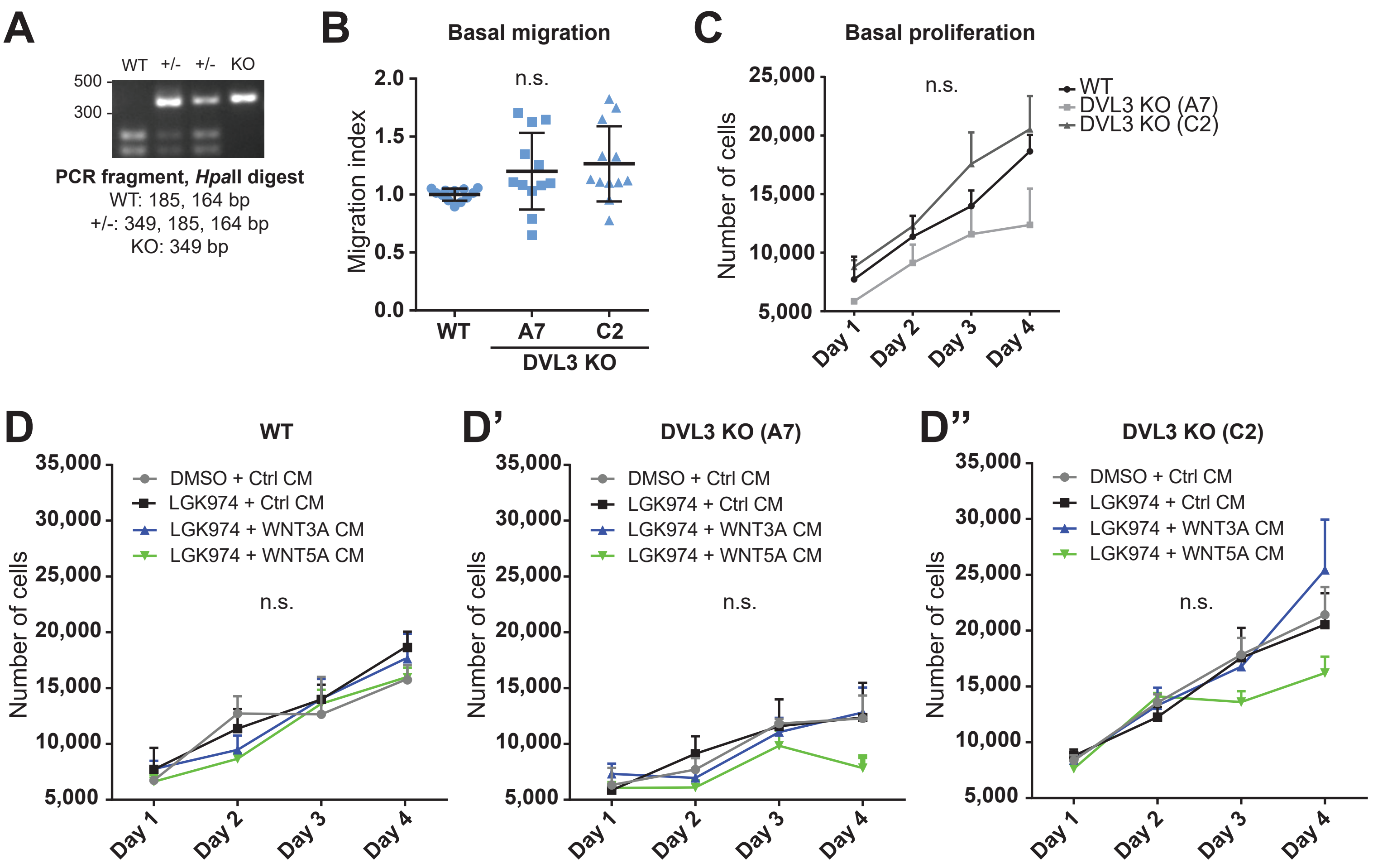

Supplementary figure S5

A

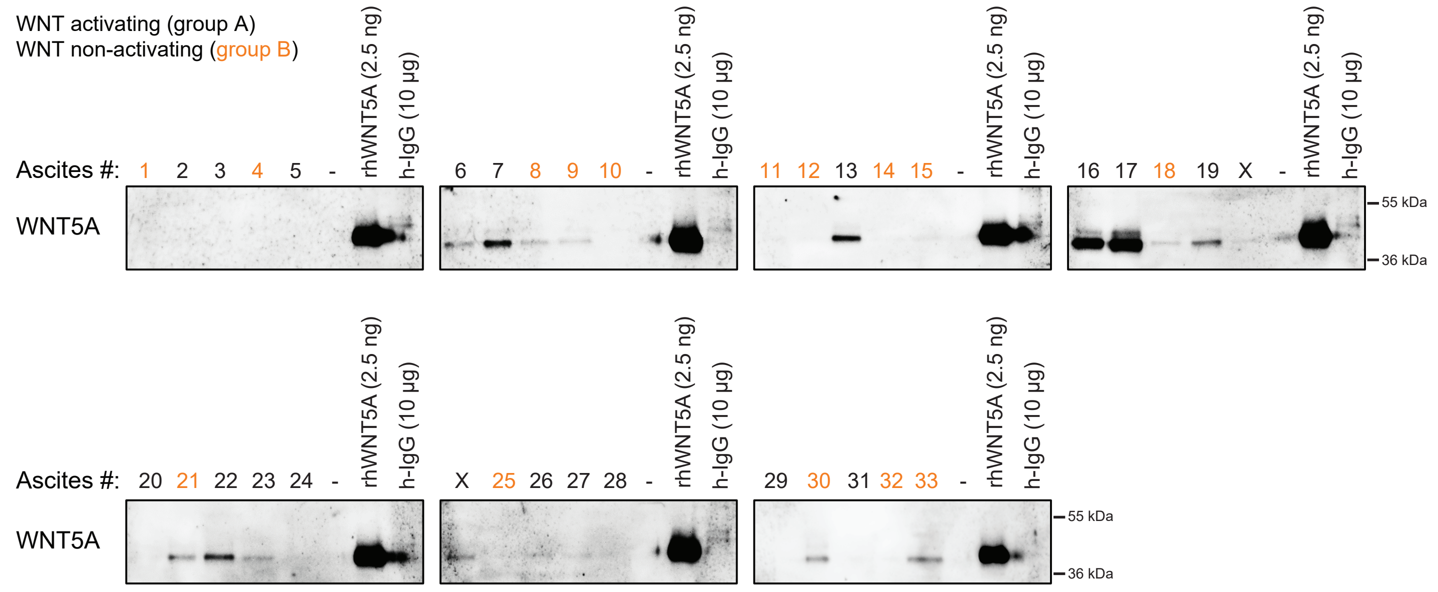

B

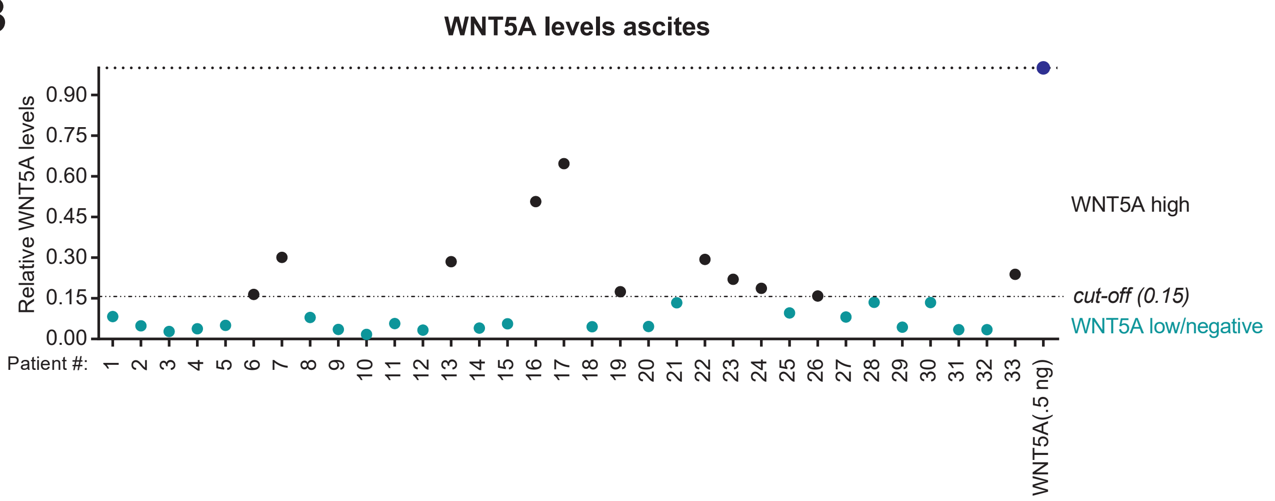

Supplementary Figure S6

A - OC vs. other cancers

Median Rank	*P* value	Gene
691.0	0.024	FZD2
844.0	0.006	ANKRD6
1162.0	0.003	VANGL2
1194.5	0.066	CELSR2
1224.0	0.032	RYK
1427.0	1.18E-11	FZD6
1456.0	0.049	PTK7
2032.0	0.011	DVL3
2897.0	0.028	CELSR1
1602.0	0.061	CSNK1A1
2356.0	0.118	WNT5A
2883.0	0.089	VANGL1
2888.0	0.104	PRICKLE1
3039.0	0.294	DVL1
3506.0	0.151	FZD3
3887.0	0.061	FZD4
4533.0	0.456	WNT11
5720.0	0.429	ROR1
6143.0	0.459	WNT4
6332.0	0.141	DVL2

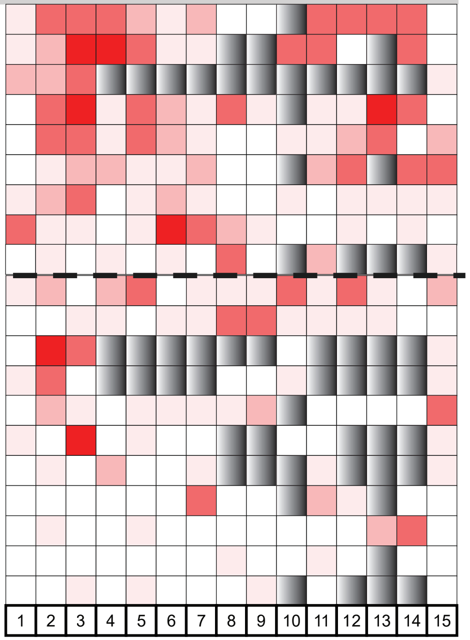

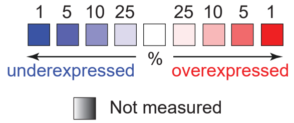

The rank for a gene is the median rank for that gene across each of the analyses. The *P* value for a gene is its *P* value for the median-ranked analysis.

B - HGSC vs. healthy ovary

Median Rank	*P* value	Gene
457.0	7.89E-7	CELSR3
548.0	4.63E-10	CELSR2
940.0	1.51E-5	FZD10
1129.0	1.75E-5	FZD2
1380.0	0.034	CSNK1D
1828.0	2.49E-6	WNT11
2191.0	0.033	PRICKLE3
2881.0	0.015	FZD6
3200.0	0.009	DVL1
3267.0	0.010	DVL3
4405.0	0.360	PTK7
5012.0	0.477	CSNK1A1
5521.0	0.117	CSNK1E
7625.0	0.954	RYK
7872.0	0.512	DVL2
8146.0	0.990	ROR2

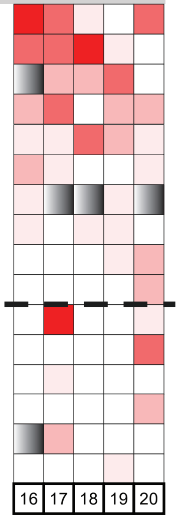

Median Rank	*P* value	Gene
1028.0	0.006	WNT5A
1260.0	1.54E-6	RYK
2491.0	0.001	FZD7
2514.0	0.030	ROR1
2516.0	0.012	CSNK1E
4262.0	0.033	ROR2
3246.0	0.305	WNT4
4333.0	0.523	CSNK1A1
5399.0	0.237	ANKRD6
5509.5	0.323	FZD4
6171.0	0.316	WNT11
6350.5	0.504	CELSR1
6820.0	0.905	CSNK1D
7473.0	0.538	DVL2
7821.0	0.731	DVL3
9508.0	0.990	FZD3

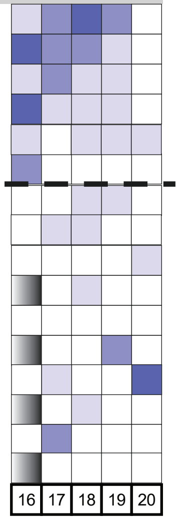

C - metastasis vs. primary site HGSC

Median Rank	*P* value	Gene
224.0	1.39E-4	CTHRC1
343.5	5.47E-4	ROR1
2045.0	0.017	VANGL1
2089.0	0.025	ROR2
2402.0	0.048	FZD7
2784.0	0.044	PRICKLE1
3309.0	0.057	FZD4
3394.5	0.078	DVL3
3706.0	0.099	FZD2

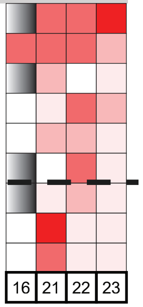

Median Rank	*P* value	Gene
256.5	7.70E-6	WNT5A
1533.0	0.005	VANGL2
2338.0	0.014	FZD10
4439.0	0.102	WNT4
4653.0	0.129	FZD6
5010.5	0.078	CSNK1E
5225.5	0.426	RYK
5813.0	0.424	CSNK1A1
5996.0	0.105	DVL1

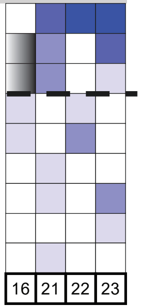

D

Original dataset reference

Number	Citation
1	*Adai CellLine, Not Published, 2008*
2	*Barretina CellLine, Nature, 2012*
3	*Bittner Multi-cancer, Not Published, 2006*
4	*Compendia CellLine, Not Published, 2007*
5	*Garnett CellLine, Nature, 2012*
6	*Gyorffy CellLine, Int J Cancer, 2006*
7	*Lee CellLine, Proc Natl Acad Sci U S A, 2007*
8	*Ramaswamy Multi-cancer, Proc Natl Acad Sci U S A, 2001*
9	*Ramaswamy Multi-cancer 2, Nat Genet, 2003*
10	*Scherf CellLine, Nat Genet, 2000*
11	*Shankavaram CellLine, Mol Cancer Ther, 2007*
12	*Shankavaram CellLine 2, Mol Cancer Ther, 2007*
13	*Staunton CellLine, Proc Natl Acad Sci U S A, 2001*
14	*Su Multi-cancer, Cancer Res, 2001*
15	*Wooster CellLine, Not Published, 2008*
16	*Adib Ovarian, Br J Cancer, 2004*
17	*Bonome Ovarian, Cancer Res, 2008*
18	*Hendrix Ovarian, Cancer Res, 2006*
19	*Lu Ovarian, Clin Cancer Res, 2004*
20	*TCGA Ovarian, No associated Paper, 2013*
21	*Anglesio Ovarian, Mol Cancer Res, 2008*
22	*Bittner Ovarian, Not Published, 2005*
23	*Tothill Ovarian, Clin Cancer Res, 2008*

Supplementary Figure S7

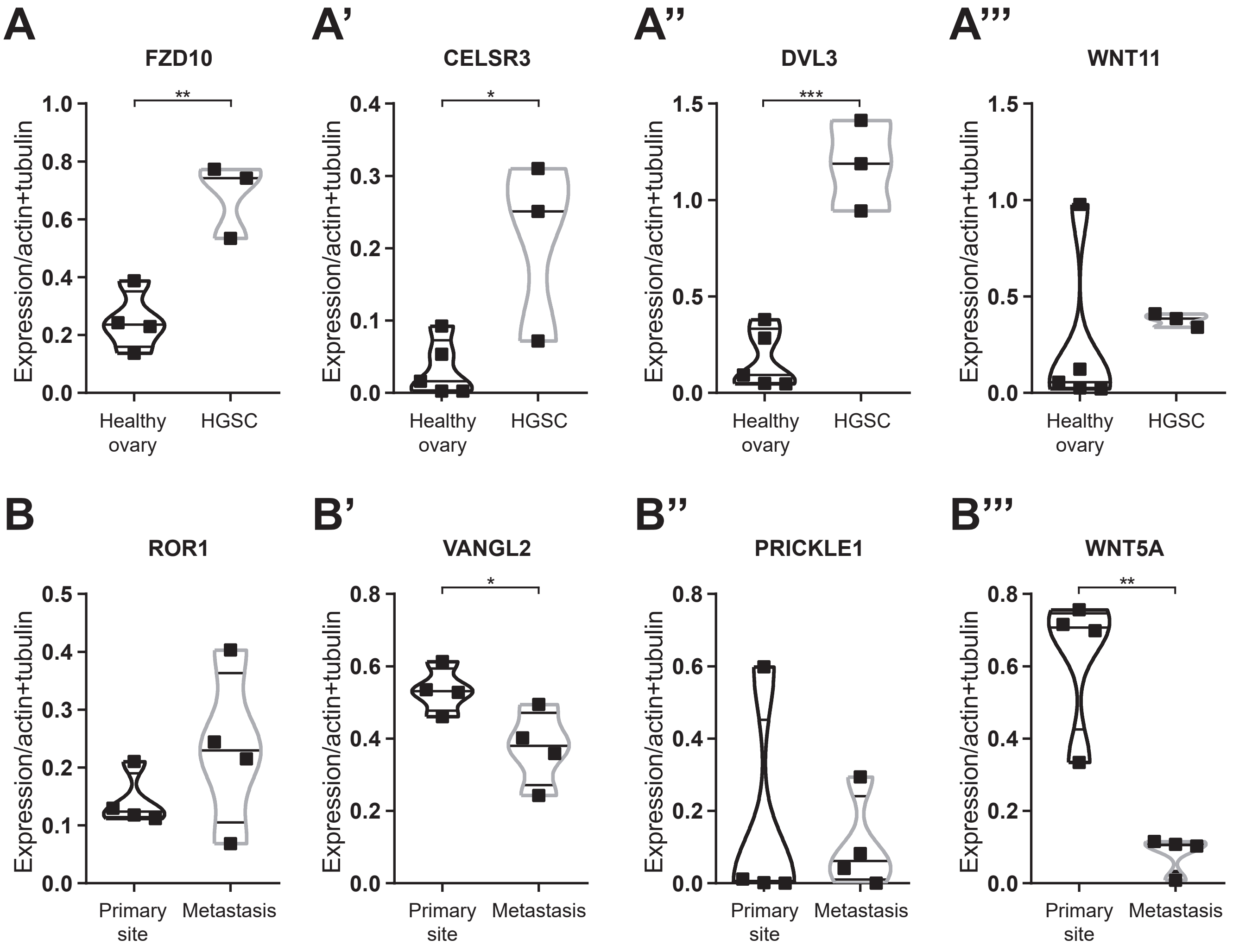

Supplementary Table ST1

Group	Activates Wnt signaling	Patient #	Age	FIGO stage (surgical)	Grade	Histology	Residual disease	BRCA mutation	1st Chemotherapy			1st Recurrence		2nd Chemotherapy			Death	
									**Regime[1]**	**End (DAD[2])**	**Progression**	**DAD[2]**	**Location[3]**	**Regime[1]**	**End (DAD[2])**	**Progression**	**DAD[2]**	**Cause**
B	no	**1**	52	IIIC	high	serous	R0	gerBRCA2 mut	P/C 3x (NACHT), P/C 3x	172	complete response	384	L, R, D	G/C/B 6x	516	progression	919	ovarian carcinoma
A	yes	**2**	72	IIIC	high	serous	>1 cm	unknown	P/C 6x	131	complete response	397	R	G/C 5x	502	complete response	692	ovarian carcinoma
A	yes	**3**	83	IVA	high	serous	>1 cm	unknown	C 6x	141	stable disease	427	L	P 24x	632	stable disease	1597	ovarian carcinoma
B	no	**4**	48	IIIC	high	serous	<1 cm	unknown	P/C 6x	131	complete response	467	L,D	PLD 5x	621	progressive disease	819	ovarian carcinoma
A	yes	**5**	51	IVB	high	serous	inoperable	unknown	P/C 4x (NACHT)	90	stable disease	-	-	-	-	-	153	ovarian carcinoma
A	yes	**6**	57	IIIC	high	serous	R0	somBRCAwt	P/C 6x	133	complete response	475	D	G/C 6x, C 3x	764	Partial response	1082	ovarian carcinoma
A	yes	**7**	58	IIIC	high	serous	R0	som BRCA1mut	P/C 5x	114	complete response	750	L, R, D	G/C 7x	953	Partial response	-	*alive*
B	no	**8***	44	IVA	high	serous	>1 cm	ger BRCAwt	G/C 6x, B	162	stable disease	1056	L, R	C 3x, cDDP 9x	1261	progression	-	*alive*
B	no	**9**	51	IIIB	high	serous	R0	unknown	P/C	195	complete response	546	R, D	C	736	unknown	782	ovarian carcinoma
B	no	**10**	57	IIIC	high	serous	R0	uknown	P/C 6x	172	complete response	389	D	-	-	-	1577	unknown
B	no	**11**	43	IIIA1	high	serous	<1 cm	unknown	P/C 4x (NACHT), P/C 2x	140	partial response	-	-	-	-	-	638	ovarian carcinoma
B	no	**12***	64	IIIC	high	serous	R0	gerBRCA1mut	C 2x, D 4x	166	complete response	781	D	cDDP	1154	stable disease	-	*alive*
A	yes	**13**	66	IIIB	high	serous	R0	uknown	P/C 6x	133	complete response	420	R, D	-	-	-	460	ovarian carcinoma
B	no	**14**	58	IIIC	high	serous	R0	unknown	P/C	141	complete response	321	R, D	T	346		493	unknown
B	no	**15**	47	IIIC	high	serous	R0	uknown	P/C 6x	147	complete response	1230	D	-	-	-	-	*alive*
A	yes	**16**	70	IIIC	high	serous	>1 cm	gerBRCAwt	P/C/B 6x	154	progressive disease	395	L	PLD 3x	455	progressive disease	687	unknown
A	yes	**17**	82	IIIC	high	serous	R0	uknown	C 9x	157	complete response	213	L, R, D	-	-	-	232	ovarian carcinoma
B	no	**18**	66	IIIC	high	serous	R0	unknown	Rejected by patient	-	-	204	L, R, D	P/C 6x	391	partial response	644	ovarian carcinoma
A	yes	**19**	67	IIIC	high	serous	R0	uknown	P/C 6x	154	complete response	340	R, D	-	-	-	403	ovarian carcinoma
A	yes	**20**	59	IIIC	high	serous	>1 cm	uknown	P/C 6x (NACHT)	161	stable disease	317	L, R	-	-	-	595	ovarian carcinoma
B	no	**21**	69	IIIA1	high	serous	R0	uknown	P/C 6x	153	complete response	-	-	-	-	-	-	*alive*
A	yes	**22**	63	IIIC	high	serous	>1 cm	gerBRCAwt	P/C/B 2x	165	partial response	319	R, D	D 3x	407	Progression	662	ovarian carcinoma
A	yes	**23**	59	IIIC	high	serous	R0	uknown	P/C 6x	138	progressive disease	153	D	P 3x, PLD 1x	211	Progression	258	ovarian carcinoma
A	yes	**24**	83	IIIC	high	serous	inoperable	uknown	C 6x	210	progressive disease	186	L, R, D	-	-	-	349	ovarian carcinoma
B	no	**25**	71	IVB	high	serous	inoperable	unknown	P/C 3x, D 4x	229	progression	239	D	-	-	-	325	ovarian carcinoma
A	yes	**26**	76	IIIC	high	serous	inoperable	uknown	P/C 10x	104	progression	120	D	G	295	progression	348	ovarian carcinoma
A	yes	**27**	68	IIIC	high	serous	R0	gerBRCAwt	P/C 6x	156	progression	182	R, D	P/B 3x	275	progression	300	ovarian carcinoma
A	yes	**28**	63	IIIC	high	serous	<1 cm	gerBRCAwt	P/C/B 6x	175	partial response	528	D	G/C	*in cursu*	-	-	*alive*
A	yes	**29**	70	IIIC	high	serous	inoperable	gerBRCA2mut	P/C 8x	223	complete response	463	R, D	C	506	progression	-	*alive*
B	no	**30**	36	IIIC	high	serous	R0	unknown	P/C 6x	146	complete response	-	-	-	-	-	-	*alive*
A	yes	**31**	72	IIIC	high	serous	inoperable	gerBRCA2 mut	P/C/B 6x	121	partial response	-	-	-	-	-	-	*alive*
B	no	**32**	71	IIIC	high	serous	>1 cm	unknown	P/C/B 6x	152	stable disease	376	D	PLD	*in cursu*	-	-	*alive*
B	no	**33**	42	IIIC	high	serous	R0	unknown	P/C 6x	153	complete response	-	-	-	-	-	-	*alive*

*ascites was collected during 1st recurrence, which was considered as a start of the study. Therefore the data about chemotherapy and recurrence in this tabhle are for 2nd and 3rd chemotherapy and 2nd recurrence.

[1] P = Paclitaxel, C = CBDCA (Carboplatin), B = Bevacizumab, G = GEM (Gemicitabine), D = Doxorubicin, PLD = Pegylated liposomal doxorubicin, cDDP (Cisplatin), T = Topotecan (Hycamtin)

[2] DAD = days after diagnosis

[3] L = local, R = regional, D = distal

Supplementary Table ST2

PCP component	Gene symbol	Gene ID	Number of mutated samples	Number of confirmed somatic non-silent	Total number of samles	% of confirmed mutated samples
ligands	WNT4	54361	2	2	673	0.30%
	WNT5A	7474	1	0	673	0.00%
	WNT11	7481	12*	3	673	0.45%
	CTHRC1	115908	1	1	673	0.15%
receptors	FZD2	2535	3	0	673	0.00%
	FZD3	7976	1	1	673	0.15%
	FZD4	8322	2	1	673	0.15%
	FZD6	8323	0	0	673	0.00%
	FZD7	8324	3	2	673	0.30%
	FZD10	11211	0	0	673	0.00%
	PTK7	5754	10*	1	693	0.14%
	ROR1	4919	6	5	693	0.72%
	ROR2	4920	3	3	693	0.43%
	RYK	6259	3	3	693	0.43%
membrane proteins	CELSR1	9620	2	1	673	0.15%
	CELSR2	1952	8	4	673	0.59%
	CELSR3	107934	14**	5	673	0.74%
	VANGL1	81839	0	0	673	0.00%
	VANGL2	57216	1	1	673	0.15%
cytoplasmic components	ANKRD6	22881	2	2	673	0.30%
	CSNK1A1	1452	0	0	693	0.00%
	CSNK1D	1453	1	0	693	0.00%
	CSNK1E	1454	3	1	693	0.14%
	DVL1	1855	1	0	673	0.00%
	DVL2	1856	0	0	673	0.00%
	DVL3	1857	0	0	673	0.00%
	PRICKLE1	144165	4	3	673	0.45%
	PRICKLE2	166336	6	2	673	0.30%
	PRICKLE3	4007	0	0	673	0.00%
	PRICKLE4	29964	1	1	673	0.15%

* please see Suppl. Table legends for explanation
** please see Suppl. Table legends for explanation

Supplementary Table ST3

	Gene_probe	cut-off	N		Events		Univariate			Multivariate		
			<cut-off	≥cut-off	<cut-off	≥cut-off	HR	95% CI	p-value	HR	95% CI	p-value
ligands	WNT4_208606	-0.951465	458	104	233	60	1.299	0.977 - 1.727	0.071			
	WNT5A_205990	2.445535	342	220	181	112	0.74	0.583 - 0.938	**0.013**	0.809	0.574 - 1.139	0.224
	WNT5A_213425	1.087275	413	149	225	68	0.725	0.552 - 0.953	**0.021**	0.859	0.580 - 1.274	0.451
	WNT11_206737	-0.902045	368	194	181	112	1.183	0.935 - 1.498	0.161			
receptors	FZD2_210220	0.808505	243	319	123	170	1.153	0.914 - 1.455	0.229			
	FZD3_219683	2.579455	470	92	250	43	0.625	0.452 - 0.865	**0.004**	0.644	0.459 - 0.905	**0.011**
	FZD4_218665	1.255265	265	297	144	149	0.756	0.601 - 0.952	**0.017**	0.850	0.668 - 1.083	0.189
	FZD6_203987	2.34851	264	298	126	167	1.106	0.877 - 1.395	0.395			
	FZD7_203705	1.368225	192	370	88	205	1.262	0.983 - 1.621	0.067			
	FZD7_203706	0.5534	110	452	47	246	1.500	1.097 - 2.051	**0.011**	1.501	1.084 - 2.08	**0.015**
	FZD10_219764	2.104765	446	116	238	55	0.656	0.489 - 0.881	**0.005**	0.695	0.509 - 0.949	**0.022**
	PTK7_207011	2.13049	293	269	139	154	1.116	0.887 - 1.404	0.348			
	ROR1_205805	0.258945	227	335	106	187	1.207	0.951 - 1.533	0.121			
	ROR1_211057	-1.22015	113	449	61	232	0.642	0.482 - 0.855	**0.002**	0.664	0.491 - 0.899	**0.008**
	ROR2_205578	-1.54917	53	509	20	273	2.059	1.304 - 3.251	**0.002**	1.597	0.992 - 2.572	**0.054**
	RYK_202853	2.96723	39	523	27	266	0.696	0.468 - 1.036	0.073			
	RYK_214172	2.23187	215	347	116	177	0.851	0.673 - 1.076	0.178			
	RYK_216976	2.142425	534	28	272	21	1.929	1.234 - 3.017	**0.003**	1.423	0.888 - 2.28	0.143
membrane proteins	CELSR1_204539	-1.741015	29	533	17	276	0.551	0.337 - 0.902	**0.016**	0.502	0.299 - 0.84	**0.009**
	CELSR1_217262	-1.327035	247	315	132	161	0.848	0.673 - 1.07	0.164			
	CELSR2_204029	1.97363	106	456	53	240	0.722	0.535 - 0.975	**0.033**	0.954	0.688 - 1.324	0.778
	CELSR2_36499	1.59268	96	466	48	245	0.781	0.572 - 1.067	0.120			
	CELSR3_205165	-0.79389	229	333	103	190	1.330	1.046 - 1.691	**0.019**	1.334	1.037 - 1.716	**0.025**
	CELSR3_40020	-0.85786	63	499	26	267	1.417	0.947 - 2.122	0.088			
cytoplasmic components	ANKRD6_204671	2.421605	280	282	148	145	0.867	0.689 - 1.091	0.224			
	ANKRD6_204672	2.183555	500	62	271	22	0.629	0.407 - 0.972	**0.035**	0.805	0.514 - 1.261	0.344
	CSNK1A1_206562	4.552265	373	189	178	115	1.266	1 - 1.602	**0.050**	1.261	0.985 - 1.614	0.066
	CSNK1A1_208865	4.918165	409	153	206	87	1.247	0.968 - 1.606	0.087			
	CSNK1A1_213086	4.643735	476	86	241	52	1.258	0.931 - 1.7	0.135			
	CSNK1A1_213860	5.01709	314	248	154	139	1.204	0.957 - 1.516	0.114			
	CSNK1D_207945	2.946665	342	220	188	105	0.695	0.546 - 0.885	**0.003**	0.739	0.571 - 0.957	**0.022**
	CSNK1D_208774	2.890305	96	466	53	240	0.693	0.514 - 0.934	**0.015**	0.749	0.544 - 1.030	0.076
	CSNK1E_202332	2.02839	330	232	155	138	1.208	0.959 - 1.521	0.108			
	CSNK1E_222015	-0.15933	219	343	127	166	0.786	0.623 - 0.991	**0.042**	0.875	0.681 - 1.124	0.296
	DVL1_203230	2.0567	510	52	274	19	0.544	0.341 - 0.868	**0.009**	0.602	0.371 - 0.976	**0.040**
	DVL2_218759	-0.842445	187	375	105	188	0.845	0.665 - 1.073	0.166			
	DVL2_57532	1.641215	419	143	224	69	0.788	0.601 - 1.034	0.085			
	DVL3_201907	-0.662335	107	455	65	228	0.628	0.476 - 0.83	**0.001**	0.613	0.459 - 0.819	**0.001**
	DVL3_201908	2.989755	199	363	115	178	0.833	0.659 - 1.054	0.128			

Supplementary Table ST4

Dataset reference	Name of dataset	Gene Expression Omnibus	Link to dataset information	Original published dataset	Note
1	Adai CellLine	GSE10843	https://www.ncbi.nlm.nih.gov/geo/query/acc.cgi?acc=GSE10843.	Stinson S, Lackner MR, Adai AT, Yu N, Kim HJ, O'Brien C, et al. TRPS1 targeting by miR-221/222 promotes the epithelial-to-mesenchymal transition in breast cancer. Science signaling. 2011; 4: ra41.	
2	Barretina CellLine	GSE36139	https://www.ncbi.nlm.nih.gov/geo/query/acc.cgi?acc=GSE36139.	Barretina J, Caponigro G, Stransky N, Venkatesan K, Margolin AA, Kim S, et al. The Cancer Cell Line Encyclopedia enables predictive modelling of anticancer drug sensitivity. Nature. 2012; 483: 603-7.	
3	Bittner Multi-cancer	GSE2109	https://www.ncbi.nlm.nih.gov/geo/query/acc.cgi?acc=GSE2109.	-	
4	Compendia CellLine	-	-	-	Compendia CellLine is a hybrid study that utilizes gene expression data on the NCI-60 cell lines as reported by Shankavaram et al., combined with sample data curated by Compendia Bioscience. This effort began in 2007 with the addition of drug sensitivity data reported by the National Cancer Institute, followed by differential expression analysis of sensitive and resistant classes for each drug. In 2008 this study was further developed by the addition of the mutation status of 27 genes across the cell line panel, as reported by the Sanger Institute. Replicates for cell lines MCF7 and IGROV1 are not included in analyses.
5	Garnett CellLine	-	https://www.ebi.ac.uk/arrayexpress/experiments/E-MTAB-783/	Garnett MJ, Edelman EJ, Heidorn SJ, Greenman CD, Dastur A, Lau KW, et al. Systematic identification of genomic markers of drug sensitivity in cancer cells. Nature. 2012; 483: 570-5.	
6	Gyorffy CellLine	GSE11812	http://www.ncbi.nlm.nih.gov/geo/query/acc.cgi?acc=GSE5846.	Gyorffy B, Surowiak P, Kiesslich O, Denkert C, Schafer R, Dietel M, et al. Gene expression profiling of 30 cancer cell lines predicts resistance towards 11 anticancer drugs at clinically achieved concentrations. Int J Cancer. 2006; 118: 1699-712.	
7	Lee CellLine	GSE5846	http://www.ncbi.nlm.nih.gov/geo/query/acc.cgi?acc=GSE5846.	Lee JK, Havaleshko DM, Cho H, Weinstein JN, Kaldjian EP, Karpovich J, et al. A strategy for predicting the chemosensitivity of human cancers and its application to drug discovery. Proc Natl Acad Sci U S A. 2007; 104: 13086-91.	
8	Ramaswamy Multi-cancer	-	http://www-genome.wi.mit.edu/cgi-bin/cancer/publications/pub_paper.cgi?mode=view&paper_id=61.	Ramaswamy S, Tamayo P, Rifkin R, Mukherjee S, Yeang CH, Angelo M, et al. Multiclass cancer diagnosis using tumor gene expression signatures. Proc Natl Acad Sci U S A. 2001; 98: 15149-54.	
9	Ramaswamy Multi-cancer 2	-	http://www-genome.wi.mit.edu/cgi-bin/cancer/publications/pub_paper.cgi?mode=view&paper_id=80.	Ramaswamy S, Ross KN, Lander ES, Golub TR. A molecular signature of metastasis in primary solid tumors. Nat Genet. 2003; 33: 49-54.	
10	Scherf CellLine	-	http://discover.nci.nih.gov/datasetsNature2000.jsp.	Scherf U, Ross DT, Waltham M, Smith LH, Lee JK, Tanabe L, et al. A gene expression database for the molecular pharmacology of cancer. Nat Genet. 2000; 24: 236-44.	
11	Shankavaram CellLine	GSE5720	http://www.ncbi.nlm.nih.gov/geo/query/acc.cgi?acc=GSE5720.	Shankavaram UT, Reinhold WC, Nishizuka S, Major S, Morita D, Chary KK, et al. Transcript and protein expression profiles of the NCI-60 cancer cell panel: an integromic microarray study. Molecular cancer therapeutics. 2007; 6: 820-	
12	Shankavaram CellLine 2	GSE5949	http://www.ncbi.nlm.nih.gov/geo/query/acc.cgi?acc=GSE5949.	Reinhold WC, Reimers MA, Lorenzi P, Ho J, Shankavaram UT, Ziegler MS, et al. Multifactorial regulation of E-cadherin expression: an integrative study. Molecular cancer therapeutics. 2010; 9: 1-16.	
13	Staunton CellLine	-	http://www.broad.mit.edu/mpr/NCI60/NC160.html.	Staunton JE, Slonim DK, Coller HA, Tamayo P, Angelo MJ, Park J, et al. Chemosensitivity prediction by transcriptional profiling. Proc Natl Acad Sci U S A. 2001; 98: 10787-92.	
14	Su Multi-cancer	-	http://www.gnf.org/cancer/epican.	Su AI, Welsh JB, Sapinoso LM, Kern SG, Dimitrov P, Lapp H, et al. Molecular classification of human carcinomas by use of gene expression signatures. Cancer Res. 2001; 61: 7388-93.	
15	Wooster CellLine	-	https://array.nci.nih.gov/caarray/project/details.action?project.experiment.publicIdentifier=woost-00041.	-	
16	Adib Ovarian	-	-	Adib TR, Henderson S, Perrett C, Hewitt D, Bourmpoulia D, Ledermann J, et al. Predicting biomarkers for ovarian cancer using gene-expression microarrays. Br J Cancer. 2004; 90: 686-92.	

17	Bonome Ovarian	-	-	Bonome T, Levine DA, Shih J, Randonovich M, Pise-Masison CA, Bogomolniy F, et al. A gene signature predicting for survival in suboptimally debulked patients with ovarian cancer. Cancer Res. 2008; 68: 5478-86.	
18	Hendrix Ovarian	GSE6008	http://www.ncbi.nlm.nih.gov/geo/query/acc.cgi?acc=GSE6008.	Hendrix ND, Wu R, Kuick R, Schwartz DR, Fearon ER, Cho KR. Fibroblast growth factor 9 has oncogenic activity and is a downstream target of Wnt signaling in ovarian endometrioid adenocarcinomas. Cancer Res. 2006; 66: 1354-62.	
19	Lu Ovarian	-	-	Lu KH, Patterson AP, Wang L, Marquez RT, Atkinson EN, Baggerly KA, et al. Selection of potential markers for epithelial ovarian cancer with gene expression arrays and recursive descent partition analysis. Clin Cancer Res. 2004; 10: 3291-300.	
20	TCGA Ovarian	-	http://tcga-data.nci.nih.gov/tcga/.	-	
21	Anglesio Ovarian	GSE12172	http://www.ncbi.nlm.nih.gov/geo/query/acc.cgi?acc=GSE12172.	Anglesio MS, Arnold JM, George J, Tinker AV, Tothill R, Waddell N, et al. Mutation of ERBB2 provides a novel alternative mechanism for the ubiquitous activation of RAS-MAPK in ovarian serous low malignant potential tumors. Mol Cancer Res. 2008; 6: 1678-90.	
22	Bittner Ovarian	GSE2109	http://www.ncbi.nlm.nih.gov/geo/query/acc.cgi?acc=GSE2109.	-	
23	Tothill Ovarian	GSE9899	http://www.ncbi.nlm.nih.gov/geo/query/acc.cgi?acc=GSE9899.	Tothill RW, Tinker AV, George J, Brown R, Fox SB, Lade S, et al. Novel molecular subtypes of serous and endometrioid ovarian cancer linked to clinical outcome. Clin Cancer Res. 2008; 14: 5198-208.	
